# Mitochondrial Quality Control in Neurodegeneration and Cancer: A Common Denominator, Distinct Therapeutic Challenges

**DOI:** 10.3390/ijms26178693

**Published:** 2025-09-06

**Authors:** Agnieszka Dominiak, Elżbieta Gawinek, Agnieszka Anna Banaszek, Anna Wilkaniec

**Affiliations:** 1 Department of Biochemistry and Pharmacogenomics, Medical University of Warsaw, 02-097 Warsaw, Poland; agnieszka.dominiak@wum.edu.pl; 2 Mossakowski Medical Research Institute, Department of Cellular Signalling, Polish Academy of Sciences, Pawińskiego 5, 02-106 Warsaw, Poland

**Keywords:** mitochondrial unfolded protein response (mtUPR), mitophagy, neurodegenerative diseases, cancer

## Abstract

Mitochondrial quality control (MQC) mechanisms, including proteostasis, mitophagy, mitochondrial dynamics, and biogenesis, are essential for maintaining mitochondrial function and overall cellular health. Dysregulation of these systems is a common feature of both neurodegenerative diseases and cancer, but the outcomes differ. Neurons depend strongly on healthy mitochondria and are easily damaged when MQC fails, resulting in organellar dysfunction and oxidative stress. By contrast, cancer cells often adapt by using MQC pathways to sustain survival and resist cell death. The mitochondrial unfolded protein response (mtUPR) and mitophagy are central to these processes, yet their roles are context-dependent. In neurodegeneration, activation of these pathways may help neurons survive, yet persistent stimulation can shift towards harmful effects. In cancer, these same pathways enhance metabolic flexibility, promote resistance to treatment, and support tumor progression. Although therapeutic strategies targeting MQC are being explored, their translation to the clinic is difficult, partly due to opposite effects in different diseases. The observed inverse epidemiological link between cancer and neurodegeneration may also reflect the distinct regulation of MQC pathways. A clearer understanding of these mechanisms is needed to identify new treatment strategies for disorders that are clinically distinct but share common mitochondrial defects.

## 1. Mitochondrial Homeostasis: From Physiological Function to Dysfunction

Mitochondria are classically recognized as the primary producers of energy in the form of adenosine triphosphates (ATPs), which are utilized in the biosynthesis of molecules critical for cell survival, growth, and reproduction. In addition to housing the tricarboxylic acid cycle and oxidative phosphorylation, mitochondria provide numerous intermediates, as well as functioning as a scaffold for metabolic pathways, including amino acid synthesis or degradation, oxidation of fatty acids, iron–sulfur cluster biogenesis, nucleotide metabolism, and partial urea synthesis [1]. Beyond these bioenergetic and metabolic roles, mitochondria are also responsible for the regulation of cell death, the metabolism of lipids, nucleotides, and amino acids, and the maintenance of calcium homeostasis [2]. These multifaceted roles make mitochondrial performance tightly linked to cellular energy demand, a dependency especially evident in neurons and rapidly dividing tumor cells.

The number of mitochondria in cells depends on energy demand and can range from a single large organelle to thousands. The nervous system consumes a significant amount of energy, and a substantial amount of energy is required to develop a rapidly dividing pathological mass, such as a cancerous tumor. Unlike Warburg’s conception that cancer’s mitochondria are dysfunctional, tumor cells rely more on mitochondrial functions than initially believed. During malignant transformation in leukemias, lymphomas, and pancreatic ductal adenocarcinoma, alterations in mitochondrial metabolic mode have been identified, leading to the upregulation of oxidative phosphorylation [3].

As semi-autonomous organelles, mitochondria contain a pool of independent DNA (mitochondrial DNA, mtDNA). In humans, mtDNA is a double-stranded, circular molecule that comprises 16,569 base pairs. Human mtDNA encodes genetic information for only 13 of about 1500 mitochondrial polypeptides, mainly proteins of respiratory enzyme complexes. Notably, mutations in mtDNA can significantly disrupt energy production and can lead to mitochondrial dysfunction and cytopathies. There are catalogs of somatic and germline mtDNA alterations in human cancers, in which a high number of truncating mutations are reported in tumorigenesis in kidney, colorectal, and thyroid cells [4,5,6].

However, the majority of outstanding mitochondrial proteins are encoded in the nuclear genome. After transcription in the nucleus, messenger RNA (mRNA) is translated by cytosolic ribosomes to produce approximately 99% of mitochondrial proteins. In contrast, the translation of proteins encoded by mitochondrial DNA occurs on mitoribosomes. Hence, there is a need to coordinate gene expression, both in the nucleus and in mitochondria, and for efficient import of mitochondrial proteins from the cytosol [7]. Within the mitochondria, synthesized polypeptide chains are folded to make a native structure of proteins. Certain proteins do not complete the folding process, and as a result, they adopt a misfolded conformation and make nonfunctional, more hydrophobic, and easily aggregating forms. In addition, under various stressors or gene mutations, there is a possibility of converting a folded protein to a misfolded one. Alzheimer’s and other neurodegenerative diseases are characterized by the accumulation of incorrectly folded proteins forming in the brain insoluble fibrils of amyloid beta (Aβ), phosphorylated tau, or α-synuclein [8,9,10,11].

Maintenance of the electrochemical potential generated during proton pumping from the matrix into the intermembrane space is associated with additional functions of mitochondria, such as ion homeostasis and the Ca^2+^ signaling pathway. In physiology, during electron transport in the mitochondrial chain at complexes I and III, electron leakage is observed. Oxygen is the terminal electron acceptor, and as a result of its incomplete reduction, superoxide (O_2_^−^) is generated. In turn, O_2_ can be dismutated to hydrogen peroxide (H_2_O_2_), which, in the Fenton reaction, generates one of the most reactive oxygen species, hydroxyl radicals. Radicals at low levels can act as second messengers that mediate intracellular signaling networks and control gene expression, but in excess, they can cause oxidative stress that damages cellular components [12].

Due to limited regulation of mtDNA machinery repair, the susceptibility of mtDNA is higher to oxidative damage than DNA in the nucleus [13]. A shifted balance between reactive oxygen species (ROS) formation and activity of antioxidative defense has been implicated in the progression of neurodegenerative diseases, like Alzheimer’s (AD) and Parkinson’s disease (PD), and is also accompanied by cancer development [14]. Mutations in mitochondrial protein-coding genes contribute to the increase in ROS levels in cancer cells. To maintain a high rate of proliferation, cancer cells demand high levels of ROS, while ROS promotes oxidation of proteins and their misfolding.

To maintain mitochondrial functionality that is critical for cellular health, a sophisticated quality control (QC) system was developed. This system consists of different types of molecular chaperones and related ATP-dependent proteolytic enzymes. QC is responsible for monitoring and degrading unfolded proteins, increasing the polypeptide repair capacity, or eliminating terminally damaged mitochondria via a dedicated form of autophagy. Deregulations in the efficiency of mitochondrial QC are now widely accepted as a contributing factor or a cause of pathological processes in neurodegenerative disorders and cancer development. The relationship between Parkinson’s disease and cancer might suggest a common pathway occurring in both diseases [15]. This dual involvement of mitochondria highlights their central role at the intersection of cellular survival and pathology.

## 2. Cellular Mechanisms of Mitochondrial Quality Control

### 2.1. The Mitochondrial Unfolded Protein Response (mtUPR): Mechanisms and Regulation

Mammalian mitochondria harbor a small circular genome of approximately 16 kb, which encodes 13 essential subunits of the electron transport chain and ATP synthase, together with rRNAs and tRNAs required for mitochondrial translation [16]. Consequently, the vast majority of mitochondrial proteins are nuclear-encoded, synthesized in the cytosol, and subsequently imported into the organelle. These proteins are produced as precursors and maintained in an unfolded, translocation-competent state [17]. To prevent their premature degradation or aggregation, they form transient complexes with cytosolic chaperones, most prominently heat shock protein 70 (HSP70) and heat shock protein 90 (HSP90) [18].

Most mitochondria-destined proteins have N-terminal presequences that are recognized by receptors on the translocase of the outer membrane (TOM) complex, which mediates their passage into the intermembrane space. The precursor protein is then transferred to the translocase of the inner membrane 23 (TIM23) to the matrix [19], where protein co-translational and post-translational folding, maturation, and degradation occur [20]. To prevent misfolding or aggregation of precursor proteins during their mitochondrial import, the translocation process is regulated by a specific group of molecular chaperones [21]. Among these chaperones, mtHSP70 (mitochondrial heat shock 70 kDa protein; also referred to as mortalin) constitutes the central component of the mitochondrial import motor, linking the TIM23 translocase to the matrix. Beyond its role in translocation, mtHSP70 cooperates with heat shock protein 60 (HSP60), a key folding chaperonin, to ensure the proper maturation of newly imported proteins [22,23]. The HSP60 and heat shock protein 10 (HSP10) form two stacked rings in the mitochondrial matrix that enclose unfolded polypeptides, assisting their ATP-dependent refolding. Tumor necrosis factor receptor-associated protein 1 (TRAP1), a mitochondrial HSP90 family member, also contributes to maintaining proteostasis and preventing ROS-dependent apoptosis [24,25]. Additionally, in the intermembrane space (IMS), small inner membrane (TIM) chaperones guide hydrophobic precursors between membrane complexes [26].

**Figure 1:**
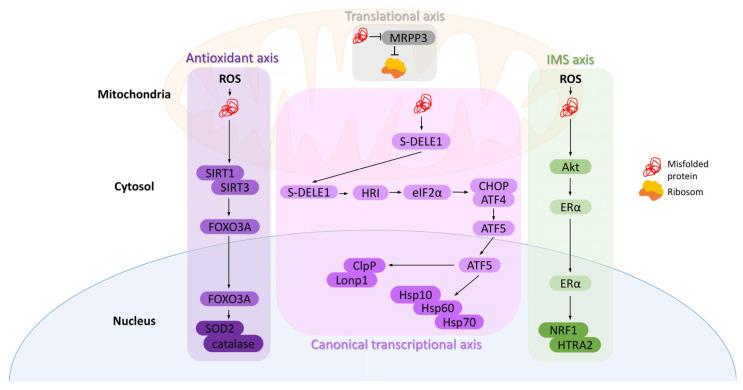
Axes of the mitochondrial unfolded protein response (mtUPR). (1) Canonical transcriptional axis: misfolded proteins in the matrix activate DELE1–HRI–eIF2α signaling, increasing CHOP, ATF4, and ATF5 activity, which induces chaperones (Hsp10, Hsp60, Hsp70) and proteases (Lonp1, ClpP). (2) Translational axis: high protein load reduces MRPP3, leading to decreased mitochondrial translation. (3) IMS axis: oxidative stress in the intermembrane space activates Akt and Erα, promoting NRF1 and HTRA2 transcription. (4) Antioxidant axis: matrix oxidative stress activates SIRT1/3, leading to FOXO3A deacetylation and nuclear translocation, inducing antioxidants like SOD2 and catalase.

However, during stress, such as oxidative stress, perturbation of the electron transport chain can lead to the accumulation of misfolded proteins, resulting in the deregulation of mitochondrial functioning [27]. To prevent that, mitochondria activate an adaptive signaling network known as the mitochondrial unfolded protein response (mtUPR) [28] (Figure 1).

The mtUPR was first characterized in rat hepatoma cells by the Hoogenraad group as a stress-induced communication pathway between mitochondria and the nucleus [29]. It results in the transcriptional upregulation of mitochondrial chaperones and proteases encoded by nuclear DNA [30]. Although most extensively studied in *Caenorhabditis elegans* [31], mammalian mtUPR has also been increasingly characterized and is known to involve at least four distinct and complementary signaling axes [32].

The accumulation of misfolded proteins within the matrix activates the canonical transcriptional mtUPR. During mitochondrial stress, the short form of DAP3-binding cell death enhancer 1 (DELE1) accumulates in the cytosol, triggering the kinase heme-regulated inhibitor (HRI), which phosphorylates eukaryotic translation initiation factor 2A (eIF2α). This phosphorylation increases the expression of C/EBP homologous protein (CHOP) and activating transcription factor 4 (ATF4). CHOP and ATF4, once phosphorylated, activate transcription factor 5 (ATF5), whose mitochondrial import is subsequently inhibited, resulting in its accumulation in the cytoplasm and translocation into the nucleus. This leads to increased expression of mitochondrial chaperones, such as HSP10, HSP60, and mtHSP70, as well as proteases Lon peptidase 1 (Lonp1) and caseinolytic protease P (ClpP) [8]. Activated HSP60 helps maintain the mitochondrial respiratory chain, and alongside HSP10 and ATP, forms a complex that encapsulates misfolded or unfolded proteins, leading to their proper folding or proteostasis. Additionally, mtHSP70 helps refold unfolded proteins. On the other hand, when chaperones fail, protease Lonp1 degrades misfolded proteins or their aggregates into short peptides, whereas ClpP maintains misfolded or damaged proteins [33].

An alternative mechanism to counteract protein misfolding is to decrease the overall protein load by reducing either translation or the import of proteins into mitochondria. Munch and Harper demonstrated the existence of another mtUPR axis—the translational axis—which regulates the folding burden inside mitochondria. A decrease in mitochondrial RNase P protein 3 (MRPP3) transcription and protein levels has been observed, resulting in a reversible reduction in mitochondrial translation [34]. Interestingly, unlike the transcriptional axis, this mechanism can be activated independently in each damaged mitochondrion, functioning as a local first-line defense mechanism [8].

Mitochondrial stressors localized in the intermembrane space (IMS) trigger yet another mtUPR axis—the intermembrane space UPR. Increased levels of ROS within the IMS activate protein kinase B (PKB, also known as Akt), which phosphorylates and activates estrogen receptor α (ERα). This, in turn, enhances the transcription of nuclear respiratory factor 1 (NRF1), promoting mitochondrial biogenesis, and the protease OMI(HTRA2) [35], which recognizes hydrophobic stretches exposed by misfolded proteins [36].

Oxidative stress within mitochondria is also a potential source of misfolded proteins. In such cases, the antioxidant mtUPR can be activated through the involvement of the sirtuin family of lysine deacetylases, primarily SIRT1 and SIRT3. These proteins regulate the activity and localization of the transcription factor forkhead box protein O3a (FOXO3A). Upon oxidative stress, SIRT1/SIRT3 are activated, leading to FOXO3A deacetylation and its translocation into the nucleus, where it stimulates the transcription of antioxidant enzymes, including catalase and mitochondrial superoxide dismutase 2 (SOD2) [20], which in turn reduces the superoxide level in mitochondria and consequently decreases oxidation and misfolding of proteins [37]. Additionally, deacetylated by SIRT3, FOXO3 coordinates mitochondrial biogenesis, fission, and fusion, as well as mitophagy [38].

However, prolonged activation of the mtUPR can lead to the propagation of damaged mitochondria, potentially exerting detrimental effects on the entire cell. In such cases, mitochondria reduce mtUPR activity and initiate mitophagy—another compensatory mechanism [39].

### 2.2. Mitophagy: Mitochondria-Selective Autophagic Clearance

For a long time, autophagy has been recognized as a random process of degrading cellular proteins and cytosolic cargo. However, much evidence suggests that the elimination of mitochondria is highly selective. Mitochondria-specific autophagy, also known as mitophagy, was first reported in 2005 by Lamasters et al. (selective degradation of mitochondria via mitophagy) [40,41].

High demand for energy and poor-quality mitochondria may enhance oxidative stress and stimulate apoptosis signals. Under these conditions, the elimination of potentially dangerous mitochondria through a specific form of autophagy may serve as a protective mechanism [1]. Dysfunctional mitochondria are degraded in a complex process that consists of initiation, priming of mitochondria for recognition by autophagy machinery, formation of the autophagosome, and at the end, lysosomal activation and hydrolytic degradation. However, the detailed mechanism of mitophagy is not fully understood.

Mitophagy is characterized by the formation of autophagosomes, which engulf mitochondria and subsequently fuse with lysosomes. The PTEN-induced putative kinase 1 (PINK1)/parkin has been recognized as a main player of mitophagy. In physiology, PINK1 is transported into the mitochondrial inner membrane and is then cleaved by proteases. The loss of mitochondrial membrane potential has been reported as a trigger of the autophagy mechanism, which stabilizes full-length PINK1 on the outer mitochondrial membrane. Putative kinase 1 is dimerized, and consequently, it phosphorylates serine 65 of pre-existing ubiquitin molecules that recruit and activate cytosolic parkin to the mitochondrial membrane. In consequence, PINK1 phosphorylates parkin and ubiquitin (Ub) to generate the catalytically active parkin–Ub complex, which is responsible for the connection of Ub chains via lysine 6 (Lys6), lysine 11 (Lys11), lysine 48 (Lys48), or lysine 63 (Lys63) to mitochondrial proteins. Labeled mitochondria by the E3 ubiquitin ligase parkin are destroyed in autophagosomes through microtubule-associated protein 1A/1B-light chain 3/GABA type A receptor-associated protein (LC3/GABARAP) interaction.

Similar to parkin, several other ubiquitin E3 ligases, including glycoprotein 78 (Gp78), SMAD-specific E3 ubiquitin protein ligase 1 (SMURF1), siah E3 ubiquitin protein ligase 1 (SIAH1), mitochondrial E3 ubiquitin protein ligase 1 (MUL1), and ariadne RBR E3 ubiquitin protein ligase 1 (ARIH1), regulate mitophagy. When localized on the mitochondrial surface, these proteins recruit adaptor proteins, including optineurin (OPTN), calcium binding and coiled-coil domain 2 (NDP52, also known as CALCOCO2), sequestosome 1 (p62/SQSTM1), neighbor of BRCA1 gene 1 (NBR1), and calcium binding and coiled-coil domain 3 (TAX1BP1, also known as CALCOCO3), which bridge the gap from damaged mitochondria through ubiquitin recognition to autophagosomes via binding to light chain 3 (LC3). Tank-binding kinase 1 (TBK1) can phosphorylate adaptor proteins and, as a result, promotes their ubiquitin binding and activates mitophagy machinery [42]. Parkin or PINK1 mutations are linked to early-onset familial Parkinsonism.

Various deubiquitinases counteract Parkin-mediated mitophagy. Among those, ubiquitin-specific protease 30 (USP30) antagonizes mitophagy [43] via removing Lys6 attached by parkin into damaged mitochondria and regulating the ubiquitin status of TOM20 (translocase of the outer mitochondrial membrane complex) [44]. A recent study shows that MF-094, a selective USP30 inhibitor, increases protein ubiquitination and accelerates mitophagy [45,46,47]. Unlike ubiquitin-specific protease 15 (USP15) and ubiquitin-specific protease 35 (USP35), ubiquitin-specific protease 8 (USP8) directly targets and stabilizes parkin to regulate mitophagy.

For many years, the mitophagy mechanism has been recognized as a ubiquitin-mediated pathway. However, a recent study revealed another category of mitophagy that is receptor- or lipid-dependent. Those alternative mitophagy models engage proteins located in the outer mitochondrial membrane as mitophagy receptors, which interact with microtubule-associated protein 1A/1B-light chain 3 (MAP1A/MAP1B LC3)-autophagosomes and recruit them to mitochondria [48].

For the parkin-independent mechanism, hypoxia can lead to an increase in the expression of autophagy mitochondrial receptors, such as fun14 domain-containing protein 1 (FUNDC1), BCL2 interacting protein 3 (BNIP3), and BCL2 interacting protein 3 like (NIX, also known as BNIP3L). Their activities are regulated by the phosphorylation status of the LC3 interaction region (LIR). FUNDC1 has been documented as a new mitophagy receptor in mammals, interacting with microtubule-associated protein light chain 3 beta (LC3B) through its LIR domain and promoting Atg5-dependent mitophagy. The phosphorylation of tyrosine 18 (Tyr18) by Src kinase reduces FUNDC1-mediated mitophagy, while under hypoxic stress, the dephosphorylation of Tyr18 stimulates the interaction between FUNDC1 and LC3B. Recent studies have revealed that BNIP3 and NIX are involved in PINK1/parkin-mediated mitophagy. Probably, the mechanism of FUNDC1-induced mitophagy differs from the pathway mediated by NIX or BNIP3. However, crosstalk between them is critical for the efficiency of energy production. Mitochondrial autophagy is a hypoxia-inducible factor 1 (HIF-1)-dependent adaptive metabolic response to hypoxia. Mutations in the LIR motif at the N-terminal region of receptors disrupt the interaction with LC3, resulting in mitophagy defects [49]. In addition to protein receptors, the lipids (ceramide and cardiolipin) can also bind LC3 and engage mitophagy. Additionally, cardiolipin can directly interact with a mitophagy regulator, Beclin1 [50].

**Figure 2:**
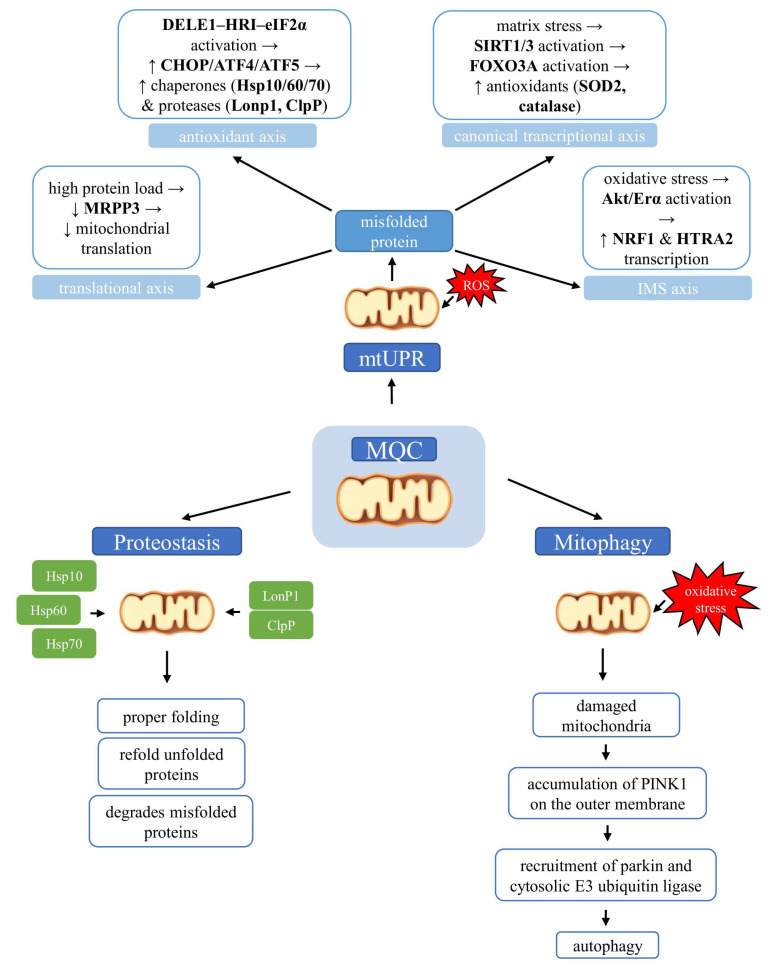
Molecular pathways of mitochondrial quality control (MQC) linking the mitochondrial unfolded protein response (mtUPR), proteostasis, and mitophagy. Accumulation of misfolded proteins within mitochondria activates the mitochondrial unfolded protein response, which operates through several axes, including transcriptional, antioxidant, intermembrane space (IMS), and translational pathways. Mitochondrial proteostasis maintains protein quality by enabling correct folding, refolding of misfolded proteins, or targeted degradation when repair is impossible. When mitochondrial damage cannot be reversed, mitophagy selectively removes dysfunctional organelles.

In summary, mitochondrial quality control encompasses a network of complementary mechanisms that safeguard organelle integrity. The mtUPR activates distinct transcriptional axes to restore proteostasis, while chaperones and proteases ensure proper folding and removal of misfolded proteins. In parallel, mitophagy selectively eliminates damaged mitochondria through the PINK1/parkin pathway and alternative routes, thereby maintaining a healthy mitochondrial pool. Together, these processes form an integrated system that continuously monitors, repairs, and renews mitochondria, enabling cells to adapt to metabolic and environmental challenges (Figure 2).

## 3. Disruption of Mitochondrial Quality Control in Neurodegeneration

### 3.1. Mitochondria as Central Players in Neuronal Vulnerability: Energy Providers or Sources of Oxidative Damage

Mitochondria play a central role in neuronal aging and neurodegeneration. Neurons, due to their high energy demands, primarily depend on mitochondrial oxidative phosphorylation (OXPHOS), which leads to increased production of ROS and heightened vulnerability to oxidative stress [51]. Limited antioxidant capacity and impaired glycolysis further exacerbate ROS accumulation, although some redirection of glucose through the pentose phosphate pathway (PPP) helps maintain redox balance [52]. Consequently, mitochondrial dysfunction contributes to hallmark features of brain aging, including dopaminergic neuron loss, glial death, and synaptic failure. These alterations, including electron transport chain (ETC) impairment, oxidative stress, disrupted calcium homeostasis, mitochondrial DNA damage, and disturbed organelle dynamics, represent converging features of several neurodegenerative conditions.

In AD, mitochondria near amyloid-β (Aβ) plaques exhibit Ca^2+^ overload, ΔΨm loss, and activation of apoptotic signaling, while both Aβ and tau contribute to a vicious cycle of ongoing mitochondrial damage [53]. In PD, reduced mitochondrial Complex I activity in the substantia nigra is accompanied by genetic defects (PINK1, PARKIN, DJ-1) and mitochondrial-targeting toxins (e.g., rotenone, MPTP) [54,55]. α-Synuclein disrupts mitochondrial membrane potential, induces fragmentation, and increases oxidative stress, partly via P2X7 receptor signaling [56,57,58,59]. In Huntington’s disease (HD), mutant huntingtin (mHTT) impairs mitochondrial transport, calcium buffering, and ATP production, increasing susceptibility to apoptosis through mitochondrial permeability transition pore (mPTP) opening [60,61]. In ALS, mutations in SOD1, TAR DNA-binding protein 43 (TDP-43), and guanine nucleotide exchange factor C9orf72 (C9orf72) affect mitochondrial bioenergetics and dynamics, with C9orf72-derived poly-GR dipeptides promoting degradation of mitochondrial ATP synthase F(1) complex subunit alpha (ATP5A1) [62,63,64,65]. During cerebral ischemia, mitochondrial depolarization, calcium overload, and excessive ROS initiate apoptotic and necrotic cascades, driven by permeability transition and cytochrome c release [66].

Importantly, mitochondrial dysfunction is also a potent driver of neuroinflammation. Mitochondrial ROS and the release of mtDNA activate innate immune pathways, including the cyclic GMP-AMP synthase-stimulator interferon genes (cGAS-STING) and toll-like receptor 9 (TLR9) signaling [67]. Microglia respond to mitochondrial stress by shifting from OXPHOS to glycolysis, increasing lactate production and proinflammatory activity [68]. This metabolic reprogramming, involving simultaneous activation of OXPHOS, glycolysis, and PPP in early stages, supports the M1 (proinflammatory) phenotype macrophage in later stages. As inflammation further impairs mitochondrial function, a self-reinforcing loop emerges that exacerbates neurodegeneration. Recent evidence indicates that defective mitochondrial quality control (MQC) is not merely a secondary outcome but an early and active contributor to neurodegeneration.

### 3.2. mtUPR in Neurodegeneration: From Protective Signaling to Detrimental Outcomes

It has been demonstrated that mitochondrial protein damage promotes the activation of mtUPR, leading to the overexpression of nuclear genes encoding mitochondrial chaperones and proteases that are imported into mitochondria in an attempt to restore protein homeostasis [28,29]. Although mtUPR activation is generally considered cytoprotective—supporting protein folding, limiting mitochondrial damage, and enhancing stress resilience—it can exert deleterious effects when overstimulated or extended. Chronic mtUPR activity has been associated with mitochondrial dysfunction and increased neuronal vulnerability, contributing to the pathogenesis of various neurodegenerative disorders [69].

In AD, both familial and sporadic forms exhibit a marked upregulation of mtUPR-associated genes and proteins, reflecting sustained activation of this pathway [70]. While such activation may initially represent an adaptive response, persistent mtUPR signaling is thought to contribute to neuronal stress and disease progression [71]. Moreover, Aβ may interact directly with mtUPR executive proteins, such as LONP1, inhibiting this protease activity, leading to disruption of mitochondrial proteostasis and dysfunction [72].

Similarly, in PD, mtUPR is implicated in the degeneration of dopaminergic neurons. In cellular models of PD, the overexpression of protective regulators, such as peroxisome proliferator-activated receptor gamma coactivator 1-alpha (PGC-1α), enhances mtUPR and mitigates oxidative stress, thereby exerting neuroprotective effects. However, it was also demonstrated that PD-related mitochondrial toxins are potent inducers of the mtUPR, thus raising the possibility that the persistent mtUPR activation may exacerbate neuronal damage [73]. Post mortem analyses of brains from PD patients harboring PINK1 mutations demonstrated an accumulation of misfolded subunits of the mitochondrial respiratory chain, accompanied by elevated expression of the mtUPR activation marker HSP60 [74].

Recently, it was demonstrated that overexpression of alpha-synuclein (α-Syn) mutants was able to induce mtUPR machinery. This induction was sustained over time, which was found to be detrimental and to cause neurodegeneration in the dopaminergic neurons of *Caenorhabditis elegans*. In addition, the co-expression of α-Syn and overactivation of mtUPR potentiated the toxicity of this protein [75].

In HD, reduced levels of mitochondrial chaperones point to a compromised mtUPR, potentially contributing to pathology [76,77]. In ALS, targeting the mtUPR may alleviate neurodegeneration and its symptoms [78]. Thus, the mtUPR exhibits a dual role—protective when transiently activated but deleterious when insufficient or chronic.

Taken together, these observations challenge the mainstream view of mtUPR as solely positive, suggesting that its effects during neurodegeneration may be either beneficial or harmful depending on the duration, intensity, and cellular environment of its activation.

### 3.3. Mitophagy in Neurodegeneration: Maintaining Fidelity or Promoting Decline?

When the mtUPR is insufficient to restore proteostasis, damaged mitochondria are typically removed via mitophagy. However, this pathway is profoundly disrupted in neurodegenerative diseases, particularly in Parkinson’s disease (PD). In both PD patients and animal models with parkin gene mutations, studies have consistently reported an accumulation of dysfunctional mitochondria and progressive neuronal degeneration [79,80]. Enhanced mitophagic activity associated with bioenergetic failure and neuronal stress has also been observed in primary cortical neurons [81] and in transgenic mice overexpressing the A53T mutated α-Syn [82]. In contrast, other investigations using transgenic models and post mortem PD brain tissue have demonstrated an accumulation of mitochondria within degenerating neurons, suggesting a failure in the mitophagic clearance phase [83].

Mechanistic insights have revealed that α-Syn can induce oxidative and nitrosative stress, promoting S-nitrosylation of parkin, which disrupts its E3 ubiquitin ligase activity and facilitates proteasomal degradation [84]. This modification has been identified as a key event in α-Syn-driven mitophagy failure. Additional findings indicate that the α-Syn-mediated decline in parkin protein levels is mainly dependent on the activation of purinergic P2X7 receptors, establishing a direct mechanistic link between extracellular α-Syn and impaired mitochondrial quality control [59].

Subsequent experiments further demonstrated that reduced parkin availability under these conditions leads to impaired parkin-dependent mitophagy and accumulation of damaged mitochondria. Notably, genetic restoration of parkin expression prevented mitochondrial dysfunction and attenuated α-Syn-induced oxidative stress, underscoring the central role of parkin in maintaining mitochondrial integrity [85]. Moreover, α-Syn-dependent stimulation of P2X7R has been shown to suppress AMP-activated protein kinase (AMPK) activity and inhibit Unc-51-like kinase 1 (Ulk1), a key initiator of the autophagy cascade [59]. This inhibition of upstream autophagy regulators suggests that α-Syn not only interferes with mitophagy execution but also disrupts autophagic initiation, further compounding mitochondrial damage. Collectively, these findings highlight the multifactorial impairment of mitophagy in PD and support the notion that extracellular α-Syn-mediated signaling contributes significantly to the collapse of mitochondrial quality control.

Defective mitophagy is also an early and causative event in AD pathology [86]. Studies using AD brain tissue, animal models, and patient-derived neurons consistently report reduced mitophagy, leading to accumulation of dysfunctional mitochondria [87]. Aβ and hyperphosphorylated tau impair PINK1-parkin-dependent mitophagy by interfering with these proteins, promoting mitochondrial fragmentation and defective clearance. This dysfunction is exacerbated by aging, which further reduces mitophagic capacity and accelerates neuronal loss. Progressive depletion of cytosolic Parkin, along with abnormal accumulation of PINK1, has been observed in AD brains and patient-derived fibroblasts [88].

Impairment of lysosomal degradation, a hallmark shared with PD, also contributes to mitophagy failure, as evidenced by autophagic accumulation and retention of mitochondria within lysosomes in AD neurons and amyloid precursor protein (APP)-mutant cell models [89,90]. Tau pathology further disrupts mitophagy through multiple mechanisms. Truncated NH2-htau fragments form stable complexes with parkin and ubiquitin-C-terminal hydrolase L1 (UCHL-1), leading to aberrant mitochondrial recruitment and impaired turnover [91]. Additionally, both human tau and its mutant form P301L have been shown to reduce parkin translocation to mitochondria in *C. elegans* and neuroblastoma cells by sequestering parkin in the cytosol, independently of ΔΨm or cytoskeletal changes [92].

These findings suggest that tau-induced mitophagy defects arise through distinct, multifaceted mechanisms. Finally, reduced basal mitophagy has been confirmed in hippocampal tissue from AD patients, induced pluripotent stem cell (iPSC)-derived cortical neurons, and AD mouse models. This impairment is associated with defective activation of ULK1 and TBK1—key initiators of autophagy and mitophagy. Notably, pharmacological enhancement of mitophagy was shown to improve memory in an AD mouse model, highlighting its therapeutic potential [86].

Furthermore, impaired mitophagy has been implicated in the denervation of neuromuscular junctions (NMJs) in ALS mouse models. In familial ALS, mutant forms of the SOD1 protein tend to form intracellular aggregates that sequester the mitophagy receptor optineurin, thereby preventing its proper incorporation into mitophagosomes. This sequestration disrupts the formation of mitophagic vesicles, reduces mitophagic flux, and leads to the accumulation of dysfunctional mitochondria. Notably, overexpression of optineurin partially restores mitophagy, underscoring its critical role in maintaining mitochondrial quality control in ALS [93]. In SOD1G93A mouse models, impaired mitophagy at NMJs is characterized by increased numbers of damaged mitochondria and decreased levels of core mitophagy-related proteins, including p62/SQSTM1, BNIP3, PINK1, and parkin. These molecular defects correlate with progressive NMJ degeneration and motor neuron loss [94]. In models of C9orf72-related ALS, loss of C9orf72 function combined with the accumulation of toxic dipeptide repeat proteins disrupts mitophagy, contributing to mitochondrial dysfunction, motor neuron apoptosis, and paralysis. Importantly, pharmacological activation of mitophagy has been shown to ameliorate motor deficits in these models, providing additional evidence for a causal role of mitophagy impairment in ALS pathogenesis [95]. However, overactivation or excessive induction of mitophagy, as seen with certain drugs like rilmenidine, can also worsen motor neuron degeneration, indicating that both insufficient and overstimulated autophagy are detrimental in ALS [96].

Similarly, defective mitophagy has been implicated in the mitochondrial abnormalities observed in HD. Under normal conditions, wild-type huntingtin (HTT) supports the assembly of autophagy-initiation complexes and the recruitment of mitophagy receptors, such as optineurin and NDP52. However, the presence of mutant huntingtin (mHTT) with an expanded polyglutamine (polyQ) tract disrupts these processes by impairing the formation of the Beclin1–Vps34 initiation complex and reducing the interaction between damaged mitochondria and autophagosomes. This leads to the accumulation of dysfunctional mitochondria, increased oxidative stress, and neuronal cell death [97]. Moreover, mHTT inactivates glyceraldehyde 3-phosphate dehydrogenase (GAPDH), a key mediator of micromitophagy, thereby inhibiting the direct lysosomal engulfment of damaged mitochondria. This defect can be partially rescued by overexpression of inactive GAPDH [98]. Additionally, mHTT recruits valosin-containing protein (VCP) to mitochondria, leading to excessive mitophagy and neuronal toxicity. Blocking the mHTT–VCP interaction normalizes mitophagy levels and reduces neurodegeneration in HD models [99,100].

Disruption of the PINK1/parkin pathway has also been reported in HD. While parkin-mediated ubiquitination of damaged mitochondria remains intact, mHTT impairs their recruitment to autophagosomes. Overexpression of PINK1 can partially restore mitophagy and improve mitochondrial function and neuronal survival in HD models [101]. Furthermore, defects in the mitochondrial–lysosomal axis and increased release of mitochondrial components via extracellular vesicles have been observed in HD, suggesting additional routes of mitochondrial quality control failure [102].

In summary, accumulating evidence underscores that mitochondrial dysfunction and the failure of quality control (QC) systems, including the mitochondrial unfolded protein response (mtUPR) and mitophagy, are not merely secondary consequences but active contributors to the pathogenesis of major neurodegenerative diseases. While initial activation of mtUPR and mitophagy may provide cytoprotective effects, their chronic dysregulation or insufficient activation can exacerbate mitochondrial damage, promote neuroinflammation, and drive neuronal loss. Converging disease-specific mechanisms across AD, PD, HD, and ALS underscore shared mitochondrial vulnerabilities, suggesting that restoring balanced QC activity could represent a promising therapeutic avenue. Altogether, these insights emphasize the dual nature of mitochondrial quality control: protective when properly balanced yet deleterious when impaired, making it a central determinant of neuronal survival or degeneration**.**

## 4. Mitochondrial Quality Control as a Determinant of Cancer Cell Survival and Proliferation

Many of the mitochondrial defects, including mtDNA depletion and mitochondrial unfolded protein accumulation, have been identified in cancer cells and suggest activation of the mtUPR mechanism. Data indicate that activation of mtUPR signaling through mitohormetic adaptation renders cancer cells more metastatic and invasive. Kenny et al. showed that patients with breast cancer with high expression of mtUPR markers (SIRT3, FOXO3a, SOD2, SOD1, LC3B, NRF1, and HSP60) have worse clinical outcomes compared to mtUPR-LOW patients [103]. The mitokines, fibroblast growth factor 21 (FGF21) and growth differentiation factor 15 (GDF15), are overexpressed during mtUPR activation and are positively correlated with hepatocellular carcinoma progression [104]. In addition, Germain’s group identified the mitochondrial deacetylase SIRT3 axes as a coordinator of the mtUPR in breast cancer [4,5]. All markers of the SIRT3 axis of the mtUPR machinery were increased in invasive cells, but their activation was not observed in normal cells [5]. However, some reports demonstrated opposite associations, linking elevated SIRT3 with prolonged survival in breast cancer [105] or poor prognosis when downregulated in pancreatic cancers [106], indicating a context-dependent role of SIRT3 in tumor metabolism [107].

Mitochondrial chaperones and proteases also contribute to tumor progression. The mitochondrial chaperone HSP60 is positively correlated with prostate tumor progression [108] and differentiation of colorectal cells [109]. In addition, mitochondrial HSP70 stimulated tumor cell survival and promoted epithelial-to-mesenchymal transition [110]. The mitochondrial-localized HSP90 and related molecule TRAP-1 are expressed at low levels in normal cells, in contrast to pancreatic and lung cancer, as well as breast adenocarcinoma, where they are intensely expressed. Repression of that pathway via HSP90 ATPase antagonists leads to selective tumor cell death [111,112]. Similarly, silencing of HSP60 triggers apoptosis in breast and colon adenocarcinoma cells via a Bax-dependent pathway [113]. Mitochondrial proteases are connected with cancer metabolic remodeling, and the loss of Lonp1 or ClpP resulted in attenuation of tumor proliferation and metastasis [114,115,116]. ClpP is overexpressed in almost every solid tumor [117]. Hence, both genetic and chemical ClpP inhibition decreased the viability of multiple acute myeloid leukemia cell lines, without effect on their regular counterparts [118].

Additionally, it was identified that the elevated level of ATF5 is found in a variety of cancers, including thyroid follicular lymphoma, epithelial ovarian carcinoma, colorectal adenocarcinoma, breast carcinoma, pancreatic cancer, and malignant glioma. Pharmacology downregulation of ATF5 function resulted in the death of breast cell lines [119,120,121,122,123].

Mitophagy in cancer represents a double-edged sword, acting either as a survival mechanism under metabolic stress or as a trigger of tumor cell death. Due to the ability of tumor cells to rapidly proliferate in the nutrient-deprived environment, cancer cells are more reliant on autophagy than their normal counterparts. The functional outcome of mitophagy is strongly dependent on cancer type and stage [124]. Early in tumorigenesis, mitophagy inhibits the formation of cancer, while in later stages, it increases cell tolerance to promote cancer development [125]. According to Guo et al.’s report, the induction of mitophagy in macrophages prevented colitis-associated cancer progression [126].

In the scenario of oxidative and metabolic stress, mitophagy removes the dysfunctional mitochondria and helps in cleaning reactive oxygen species in cancer [127]. Studies have also proved that mitophagy, by promoting the Warburg effect, can help tumor cells to escape from immune surveillance [128]. Alternatively, cancer cells can suppress autophagy as a mechanism to avoid quality control.

In general, the cytotoxicity of chemotherapeutic treatments is attributed to the enhancement of superoxide formation, induction of oxidative stress, and mitochondrial dysfunctions. Thus, the degradation of defective mitochondria by autophagy is thought to be a mechanism of drug resistance in cancer. A combination of an autophagy inhibitor with radiotherapy resulted in the enhancement of radiotherapy cytotoxicity in resistant cancer cells [129]. Moreover, inhibition of PINK1/parkin- or ras-related protein Rab-9A (Rab9a)-mediated mitophagy has been shown to radiosensitize cancer cells [130]. Similarly, genetic downregulation of key mitophagy receptors, including PINK1, FUNDC1, and activating molecules in BECN1-regulated autophagy protein 1 (AMBRA1), produced a comparable effect [131,132,133]. In turn, stimulating mitophagy in acute myeloid leukemia by mitochondria-targeted ceramide analog reduced the resistance to crenolanib [134].

There are observations that certain receptors and adaptors associated with mitophagy are down or upregulated in cancer patients. Different mutated forms of the PARK2 gene appear in cancers. Mice with parkin knockout are more prone to develop hepatic tumors [135,136]. In glioma, melanoma, breast, lung, and colon cancers, the parkin/PARK2 gene has been identified as a mutation carrier (parkin, a gene implicated in autosomal recessive juvenile Parkinsonism, has also been proposed as a candidate tumor suppressor located on chromosome 6q25–q27) [137,138,139,140,141]. Cancer-specific mutations abolish suppressive effects of the PARK2 protein by decreasing the E3 ligase activity [142]. Another study revealed that the parkin expression was increased in melanoma, while parkin deficiency induced apoptosis and suppressed the growth of melanoma tumors through the inhibition of mitofusin-2 (Mfn2) ubiquitination [143]. On the contrary, the downregulation of parkin expression stimulates proliferation and tumorigenesis in pancreatic cancer [144]. PINK1 is recognized as a negative regulator of glioblastoma growth. A low level of PINK1 expression induces ROS formation and tumor brain growth [145]. In contrast, in lung cancer, PINK1 expression is upregulated, which promotes chemoresistance and cancer proliferation [146]. Furthermore, after neoadjuvant chemotherapy in esophageal squamous cell carcinoma patients, the expression of PINK1 and LC3 was higher compared to untreated patients, and the inhibition of mitophagy restored chemosensitivity [147].

In addition, the activation of the BNIP3 receptor has a breast cancer suppressor effect, and according to Chourasia et al., BNIP3 deficiency leads to ROS production and mammary neoplastic development [148]. At the same time, the activation of BNIP3 promotes the growth of melanoma and renal cell carcinoma. Furthermore, the downregulation of BNIP3 expression in pancreatic cancer is responsible for chemoresistance and is connected with poor prognosis [149]. In turn, epigenetic silencing of BNIP3 resulted in high aggressiveness of the pancreatic cancer cells [150]. Another study confirmed that there is a correlation with the silencing of BNIP3 expression and invasiveness or metastasis in breast, hematological malignancies, gastric, pancreatic, lung, and liver cancer [151]. Conversely, an earlier study indicated that BNIP3 and NIX were upregulated in highly invasive breast ductal carcinoma [152]. Moreover, oncogenic GTPase KRas (KRAS) induces NIX-mediated mitophagy and favors the development of pancreatic cancer, but the lack of NIX results in mitochondrial function restoration and thus delays the progression of pancreatic cancer [153].

Li et al. showed that specific knockout of FUNDC1, a previously characterized mitophagy receptor, promotes tumor growth induced by diethylnitrosamine. However, FUNDC1 overexpression in transgenic hepatocytes prevents hepatocellular carcinoma development [154]. While recent studies have shown that the expression of FUNDC1 in cervical cancer cells was significantly increased and its depletion leads to inhibiting the tumor cells’ proliferation and increases cell sensitivity to chemotherapy and ionizing radiation [132].

Altogether, these observations highlight that cancer cells exploit mitochondrial quality control pathways in a highly context-dependent manner. While restoring proteostasis and mitophagy is protective in neurodegeneration, tumors can co-opt the same mechanisms for survival, therapy resistance, and metastatic progression. Thus, the patient-specific modulators of mitochondrial QC could become a breakthrough in cancer therapy, but more research and careful consideration are needed. Chloroquine and hydrochloroquine, autophagy regulators, have entered into many clinical studies on anticancer therapy, and recent reports demonstrated beneficial effects of their use (available online: https://clinicaltrials.gov/ct2/results?term=autophagy+and+cancer (accessed on 31 July 2025)). In addition to patient-specific treatment, the combination of anticancer therapies that attack the tumor at multiple levels is necessary.

## 5. Does Mitochondrial Quality Control Breakdown Represent a Converging Mechanism in Neurodegenerative Diseases and Cancer?

**Figure 3:**
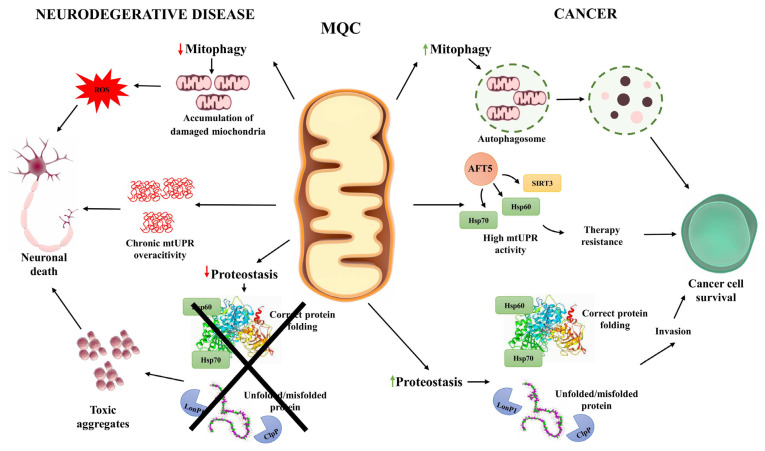
Cellular mechanisms of mitochondrial quality control (MQC) in neurodegenerative diseases and cancer. This schematic illustrates how mitochondrial quality control pathways contribute to distinct cellular outcomes in neurodegeneration and cancer. In neurodegenerative diseases, impaired mitophagy results in the accumulation of damaged mitochondria and reactive oxygen species (ROS), leading to oxidative stress and neuronal death. Chronic overactivation of the mitochondrial unfolded protein response (mtUPR) contributes to maladaptive stress, further compromising neuronal survival. In parallel, reduced proteostasis capacity prevents proper folding and repair of mitochondrial proteins, causing toxic aggregate accumulation that exacerbates neurodegeneration. Cancer cells display the opposite pattern. Mitophagy is often upregulated, promoting the removal of damaged mitochondria and supporting adaptation to hypoxic conditions. Sustained mtUPR activity, mediated by factors such as ATF5, SIRT3, and HSP60/70, enhances proteostasis, promotes stress tolerance, and underlies resistance to therapy. Elevated levels of chaperones (Hsp60, Hsp70) and proteases (LonP1, ClpP) maintain efficient folding and degradation of misfolded proteins, supporting tumor cell proliferation and invasion. Together, the figure highlights the divergent consequences of MQC dysregulation—vulnerability and cell loss in neurons versus enhanced survival and malignancy in cancer cells.

Numerous internal and external factors affect the genomic and metabolic integrity of human cells. While the consequences of defective responses to genomic damage are well documented in the context of proliferating cells and tumorigenesis, their significance in postmitotic neuronal cells remains poorly understood. Accumulating evidence reveals that both cancer and neurodegenerative diseases, despite their divergent clinical manifestations, exhibit shared molecular vulnerabilities, many of which converge on MQC systems (summarized in Figure 3).

These processes include mitochondrial dynamics, mitophagy, mtUPR, proteostasis, and biogenesis, all of which are crucial for maintaining mitochondrial homeostasis and cellular viability [4,9,155].

Mitochondrial damage mechanisms intersect with deregulated MQC pathways, generating both overlapping and disease-specific outcomes (Table 1). For instance, neurons and cancer cells are both subject to persistent oxidative stress, although their adaptive responses are notably different. In neurodegenerative diseases, such as Alzheimer’s disease and Parkinson’s disease, oxidative damage impairs mitochondrial respiration and leads to the accumulation of dysfunctional mitochondria, often accompanied by increased mtDNA deletions and impaired bioenergetics PD [156,157,158]. Conversely, cancer cells tend to exploit mitochondrial dysfunction to drive metabolic reprogramming, such as increased aerobic glycolysis, and to resist apoptotic stimuli [159,160].

Among the key MQC elements implicated in both disease types is the PINK1/parkin pathway, central to mitophagy. In neurodegeneration, loss-of-function mutations in PINK1 or parkin inhibit mitophagic clearance of damaged mitochondria, exacerbating oxidative stress and neuronal cell death [161]. In contrast, many cancers maintain functional or even elevated mitophagy to adapt to hypoxic and nutrient-deficient tumor microenvironments, facilitating tumor growth and survival [1]. Likewise, the mtUPR plays context-dependent roles in both pathologies. In AD, experimental activation of the mtUPR via doxycycline improves mitochondrial function and reduces amyloid pathology in model organisms, suggesting a cytoprotective function [162]. However, chronic or unrestrained mtUPR activation, as observed in some models of PD, can induce neuronal loss, emphasizing the hormetic nature of this pathway. In cancer, components of the mtUPR, such as ATF5, HSP60, and HSP70, are frequently upregulated, contributing to treatment resistance, proliferation, and metastatic potential [75].

**Table 1:** Comparative overview of mitochondrial damage mechanisms and MQC dysregulation in neurodegeneration versus cancer.

Category	Neurodegeneration	Cancer	References
**Mitochondrial damage mechanisms**			
mtDNA integrity	High susceptibility to oxidative damage, deletions, and mutations impairing bioenergetics, leading to progressive neuronal dysfunction.	mtDNA instability fuels metabolic reprogramming, supporting aerobic glycolysis and tumorigenesis.	[156,157,158,159,160]
Oxidative stress	Persistent ROS damages the respiratory chain, proteins, and mtDNA, triggering neuroinflammation and apoptosis.	Cancer cells tolerate higher ROS levels and use them as signaling mediators to sustain proliferation and angiogenesis.	[127,128,156,157,158]
**Mitochondria–nuclear crosstalk in DNA damage**			
	Mitochondrial dysfunction exacerbates nuclear DNA damage, promoting apoptosis in postmitotic neurons unable to re-enter the cell cycle.	Mitochondrial dysfunction promotes genomic instability that fuels proliferation and malignant transformation.	[163,164]
**MQC pathways**			
Mitophagy (PINK1/parkin, BNIP3, NIX, FUNDC1)	Loss-of-function mutations or impaired activity reduce the clearance of damaged mitochondria, exacerbating oxidative stress and neurodegeneration.	Often upregulated or rewired to adapt to hypoxia and nutrient stress, promoting tumor growth and therapeutic resistance.	[70,131,132,133,148,149,150,151,152,153,154,161]
mtUPR (ATF5, HSP60, HSP70, SIRT3 axis)	It can be neuroprotective when transiently activated, but chronic activation induces neuronal death.	Frequently upregulated, enhances survival, proliferation, metastasis, and therapy resistance.	[5,75,103,104,105,106,107,108,162]
Proteostasis (Lonp1, ClpP, chaperones)	Insufficient clearance of damaged proteins contributes to proteotoxic stress and neuronal loss.	Overactivation sustains tumor metabolism and survival; ClpP inhibition reduces cancer cell viability.	[108,109,110,111,112,113,114,115,116,117,118]
Mitochondrial dynamics (fusion/fission: mitofusin 1 and 2 (Mfn1/2), mitochondrial Dynamin-like GTPase OPA1 (OPA1), Dynamin-related protein 1 (Drp1)	Imbalanced dynamics impair mitochondrial distribution and synaptic function.	Dysregulated dynamics favor metabolic reprogramming, proliferation, and metastasis.	[4,9,115]
Biogenesis (PGC-1α, NRF1, mitochondrial transcription factor A (TFAM))	Impaired induction leads to reduced mitochondrial renewal and energy deficiency.	Often hijacked to support rapid proliferation and metabolic plasticity.	[4,9,115]

DNA damage is another shared hallmark, though with opposite consequences. While in cancer this often promotes cell cycle re-entry, genomic instability, and uncontrolled proliferation [163], in neurons it leads to apoptosis [164]. Notably, mitochondrial dysfunction may precede nuclear genomic damage. The susceptibility of mtDNA to oxidative modifications, due to its proximity to the electron transport chain and lack of protective histones, contributes significantly to both oncogenesis and neurodegeneration [157]. Moreover, non-selective voltage-gated ion channel VDAC1 (VDAC1), a mitochondrial membrane protein regulating metabolism and apoptosis, is upregulated in both neurodegeneration and many cancers but exhibits contrasting roles: pro-apoptotic interactions with misfolded proteins in AD and PD versus apoptosis suppression in malignancies [165,166].

Interestingly, epidemiological observations reveal an inverse comorbidity between cancer and certain neurodegenerative diseases. Patients with AD or PD often exhibit a lower incidence of cancer and vice versa [157]. Although not universal across all cancer types, e.g., melanoma shows increased co-occurrence with PD [167], this suggests the involvement of molecular regulators whose differential expression modulates risk in opposite directions. For instance, cellular tumor antigen p53 (p53) is upregulated in AD and PD, promoting apoptosis, whereas its loss of function in tumors permits evasion of cell death [168,169]. Similarly, peptidyl-prolyl cis-trans isomerase NIMA-interacting 1 (Pin1), which controls protein folding and function via isomerization of phosphorylated serine/threonine-proline motifs, is downregulated in AD but overexpressed in cancers [170].

These shared yet divergent molecular signatures underscore the duality of mitochondrial quality control pathways in health and disease. In neurons, failure to clear damaged mitochondria and restore proteostasis contributes to degeneration, while in tumors, partial dysfunction of these same pathways often enhances cellular fitness, supporting unchecked growth. The dual roles of MQC mechanisms like mitophagy and mtUPR necessitate disease- and context-specific modulation to achieve therapeutic benefits without exacerbating pathology.

## 6. Targeting Mitochondrial Quality Control: Therapeutic Opportunities and Future Directions

Mitochondrial quality control mechanisms, particularly mitophagy and the mitochondrial unfolded protein response (mtUPR), have emerged as critical regulators of cellular homeostasis and survival under stress conditions. Their dysregulation is now widely recognized as a common feature of numerous pathological states, notably neurodegenerative diseases and cancer. Consequently, targeting these pathways has become a promising avenue for therapeutic intervention, though not without significant complexity.

In neurodegenerative diseases, such as Alzheimer’s disease, Parkinson’s disease, amyotrophic lateral sclerosis, and Huntington’s disease, defective mitophagy leads to the accumulation of dysfunctional mitochondria, increased oxidative stress, and subsequent neuronal loss. Pharmacological agents that enhance mitophagy have demonstrated neuroprotective effects in multiple preclinical models. Compounds such as urolithin A, actinonin, and spermidine activate PINK1/parkin - and NIX-dependent mitophagy, leading to attenuation of AD-related pathology, improved mitochondrial function, and ameliorated cognitive deficits [171,172]. Similarly, nicotinamide adenine dinucleotide, oxidized form (NAD^+^) precursors, including nicotinamide riboside (NR) and nicotinamide mononucleotide (NMN), promote mitophagy through sirtuin activation and enhancement of phosphoinositide-3-kinase-protein kinase B (PI3K-Akt) and mitogen-activated protein kinases/extracellular signal-regulated kinases 1 and 2 (MAPK/ERK1/2) signaling [173]. In both AD and PD models, restoration of NAD^+^ levels has been associated with reduced neurodegeneration and preservation of mitochondrial integrity [174,175]. Other strategies, such as inducing mild bioenergetic stress with agents like 2-deoxyglucose [176] or mitochondrial uncouplers [177], or using autophagy enhancers like rapamycin and metformin, have also been shown to activate mitophagy and mitigate neurodegenerative phenotypes [178,179,180]. For example, rilmenidine improved disease progression in animal models and was well tolerated in a pilot clinical experiment (EudraCT 2009-018119-14), justifying further randomized trials to assess efficacy. Activation of autophagy to remove misfolded proteins, in ALS, is being clinically tested: trials are underway with rapamycin (NCT03359538), colchicine (NCT03693781), and tamoxifen (NCT02166944). Rapamycin is being evaluated in a Phase II trial (NCT04629495) for biological and clinical effects in AD treatment [181]. Despite numerous challenges, strategies based on selective mitophagy enhancement, including USP30 inhibitors and PINK1 activators, are entering Phase I clinical trials for the first time [182].

Notably, nilotinib, developed initially as a tyrosine kinase inhibitor, has gained attention for its ability to increase parkin abundance and activity through proteasomal recycling mechanisms and inhibition of c-Abl-mediated tyrosine phosphorylation [183]. In multiple neurodegeneration models of AD and PD, and also in preclinical studies, chronic nilotinib treatment restored mitophagy, enhanced clearance of protein aggregates, and improved motor and cognitive performance, suggesting its potential for clinical translation [184,185,186].

In contrast to the neuroprotective role of mitophagy in neurodegeneration, the same pathway may exert protumorigenic effects in cancer [133]. Cancer cells frequently exploit mitophagy to maintain metabolic flexibility, resist oxidative damage, and survive chemotherapeutic stress [18]. Accordingly, inhibition of mitophagy has emerged as a viable strategy to sensitize tumors to conventional therapies [187]. For instance, in cervical cancer, melatonin has been shown to inhibit mitophagy by downregulating c-Jun N-terminal kinase (JNK) and parkin expression, thereby enhancing the apoptotic response to cisplatin [188]. In hepatic carcinoma, suppression of DRP1-dependent mitophagy using mitochondrial division inhibitor 1 (mdivi-1) or the lysosomal inhibitor, bafilomycin, increases cellular susceptibility to chemotherapeutic agents [189]. Similarly, liensinine enhances cisplatin sensitivity in breast cancer by impairing autophagosome maturation and lysosomal hydrolase activity [190]. Conversely, in other tumor types, such as hepatocellular carcinoma, agents like ketoconazole and sorafenib paradoxically promote mitophagy through the stabilization of PINK1 and recruitment of parkin, which can culminate in apoptotic cell death [191]. Mitochondria-targeted drugs such as mitochondria-targeted carboxy-proxyl (Mito-CP) and mitochondrial targeting of metformin (Mito-metformin) have also been shown to induce mitophagy by releasing ULK1 from mammalian (mechanistic) target of rapamycin (mTOR) inhibition and lowering mitochondrial membrane potential, ultimately leading to reduced proliferation of colorectal cancer cells [192]. These findings illustrate the dualistic nature of mitophagy in cancer biology and underscore the necessity for disease- and context-specific approaches in targeting mitochondrial quality control pathways. In Table 2, we present potential therapeutics involving mitophagy inhibitors or inducers in cancer and neurodegenerative diseases.

Parallel to mitophagy, the mtUPR also represents a double-edged sword. In neurodegenerative conditions, controlled activation of mtUPR supports proteostasis and mitochondrial function, conferring cytoprotection. However, persistent or excessive mtUPR activation may exacerbate pathology by triggering inflammatory responses, promoting apoptosis, or contributing to mitochondrial DNA instability [209]. In cancer, mtUPR may allow malignant cells to adapt to hypoxic or metabolic stress and evade cell death. Inhibiting mtUPR in such contexts has been shown to reduce tumor viability and enhance sensitivity to anticancer therapies [14]. Yet, despite encouraging results from preclinical studies, there are currently no approved drugs that selectively modulate mtUPR in humans.

Translational challenges further complicate the therapeutic potential of targeting mitochondrial quality control. The context-dependent nature of mitophagy and mtUPR demands a precise therapeutic window. While enhancement of these pathways is beneficial in degenerating neurons, the same interventions may promote survival and resistance in cancer cells. Conversely, mitophagy inhibition may potentiate chemotherapeutic efficacy in cancer but risks accelerating neurodegeneration. These paradoxes highlight the need for rigorous mechanistic studies and the development of selective modulators with minimal off-target effects. Although some mitophagy-enhancing agents have entered early-phase clinical trials, particularly for neurodegenerative diseases, their clinical translation is still in its infancy. Biomarker discovery will be crucial for patient stratification and therapeutic monitoring. For example, circulating mitochondrial components, expression profiles of mitophagy, or mtUPR-related genes, and imaging-based assessments of mitochondrial function could serve as tools to predict treatment response and tailor interventions. Moreover, understanding the spatiotemporal dynamics of mtUPR and mitophagy across different disease stages and cell types could reveal new therapeutic windows and inform combination strategies.

Nevertheless, despite advances in targeting mitochondrial quality control, important questions remain regarding contradictory results, model limitations, and the lack of suitable biomarkers.

## 7. Controversies, Limitations, and Future Perspectives

Despite rapid progress in understanding MQC, many uncertainties remain. In particular, the roles of the mitochondrial unfolded protein response (mtUPR) and mitophagy are not consistent across studies and disease models. In neurons, mtUPR activation can restore proteostasis and support survival, but sustained or excessive signaling may exacerbate stress and increase vulnerability [28,29,69,70,71,72,73,74,75]. In cancer, mtUPR components often promote adaptation and resistance [5,103,104], although some reports link high SIRT3 expression with an improved outcome [105,106]. These discrepancies highlight the need to define the thresholds and regulators that determine whether mtUPR acts in a protective or harmful manner.

Mitophagy is similarly complex. In Parkinson’s disease, both insufficient and excessive mitophagy have been reported, depending on the model and stage of disease [59,79,80,81,82,83,84]. In Alzheimer’s disease, amyloid-β and tau proteins interfere with PINK1/parkin activity and lysosomal function, but the precise sequence of events leading to mitochondrial accumulation is not fully understood [86,87,88,89,90,91,92]. In cancer, mitophagy may suppress tumor initiation yet later support tumor growth and therapy resistance [124,125,126,127,128,129,130,131,132,133]. Understanding how mitophagy is shaped by cell type, microenvironment, and disease stage remains a key challenge.

Another limitation arises from the experimental models used. Most findings rely on rodent systems or cultured cells, which cannot fully reproduce the complexity of human neurons or the heterogeneity of tumors. Human-based approaches, including iPSC-derived neurons and patient-derived organoids, will be essential to bridge this gap.

Finally, there is a critical lack of biomarkers that can reliably monitor MQC activity in patients. Without such tools, it is difficult to assess pathway activity in vivo, stratify patients according to their mitochondrial status, or evaluate the effects of potential modulators. This absence not only limits mechanistic studies but also hampers clinical translation.

Future research should therefore focus on three main directions: (i) defining molecular switches that determine whether mtUPR and mitophagy act in protective or detrimental ways, (ii) developing advanced human-relevant models, and (iii) establishing robust biomarkers for MQC activity. Addressing these challenges will be essential for transforming current knowledge into effective, clinically relevant strategies.

In conclusion, mitochondrial quality control represents a central determinant of both neurodegeneration and cancer. Its dual and context-dependent nature demands precise, tailored approaches if experimental insights are to be translated into clinical benefit.

## Figures and Tables

**Table 2:** The impact of selected drugs on mitophagy in the treatment of cancer and neurodegenerative diseases.

Drug	Type of Disease	Mitophagy Inhibitor/Inducer	Direct Target	Mechanism in Therapeutics	Clinical Trial Status	Off-Target Effects/Side Effects
Ketoconazole	Cancer	Inducer	PINK1/parkin	Promotes apoptosis of liver cancer cells and induces the PINK1-parkin mitophagy pathway [191]	No	severe hepatotoxicity (FDA black box warning); adrenal insufficiency (inhibition of adrenal steroidogenesis); strong CYP3A4 inhibition → extensive drug–drug interactions [193]
Sorafenib (BAY 43-9006)	Cancer	Inducer	PINK1	Stabilizes PINK1 on the outer membrane of the mitochondria [194]	A Phase II Study of BAY 43-9006 (Sorafenib) in Metastatic, Androgen-Independent Prostate Cancer ClinicalTrials.gov ID NCT00090545	hypertension, diarrhea, hand–foot skin reaction [195]
Liensinine	Cancer	Inhibitor	Dynamin-1-like protein (DNM1L)	Suppresses the fusion of the autophagosome with the lysosome and the excessive accumulation of autophagosomes [190]	No	cardiotoxicity, antiarrhythmic effects at high doses [196,197]
Melatonin	Cancer	Inhibitor	JNK/parkin	Enhances apoptosis of cells and lowers the level of parkin and c-Jun N-terminal kinase (JNK) [188]	Phase I Dose Finding Study for Melatonin in Pediatric Oncology Patients with Relapsed Solid Tumors ClinicalTrials.gov ID NCT01858155	drowsiness, headache, endocrine disruption (rare) [198,199]
Mdivi-1	Cancer	Inhibitor	DRP1	Inhibits DRP1-mediated mitophagy and enhances cisplatin-induced apoptosis	No	poor specificity, off-target inhibition of mitochondrial respiration [200,201]
Urolithin A, actinonin, spermidine	Neurodegenerative disease	Inducer	PINK1/parkin	Activates PINK1/parkin- and NIX-dependent mitophagy [171]	No	gastrointestinal discomfort, interindividual variability [202]
Resveratrol	Neurodegenerative disease	Inhibitor	PINK1/parkin	Weakens the overexpression of PINK1/parkin, inhibiting mitophagy and restoring mitochondrial homeostasis	No	poor bioavailability, drug interactions [203,204]
Rapamycin	Neurodegenerative disease	Inducer	mTOR	Activates mitophagy and enhances lysosomal biogenesis and autophagosome formation [181] Activates mitophagy and its downstream PINK1-parkin pathway [197]	Multiple trials: HD (preclinical evidence), ALS (NCT03359538), AD (Phase II, NCT04629495)	immunosuppression, dyslipidemia, insulin resistance [205,206]
Metformin	Neurodegenerative disease	Inducer	Complex I of ETC	Inhibits mTORC1 through AMPK and the TSC complex [196] Decreases ER stress and p53 expression, resulting in induction of parkin-mediated mitophagy [198]	Preventing Cognitive Decline with Metformin ClinicalTrials.gov ID NCT04511416	gastrointestinal upset, lactic acidosis (rare) [207,208]
Nicotinamide riboside	Neurodegenerative disease	Inducer	Sirtuins	Promotes mitophagy through sirtuin activation and enhancement of PI3K-Akt and MAPK/ERK1/2 signaling [191]	Metabolic Cofactor Supplementation in AD and PD Patients ClinicalTrials.gov ID NCT04044131 A Randomized Controlled Trial of Nicotinamide Riboside Supplementation in Early Parkinson’s Disease ClinicalTrials.gov ID NCT03568968 N-DOSE: A Dose Optimization Trial of Nicotinamide Riboside in Parkinson’s Disease ClinicalTrials.gov ID NCT05589766	flushing, liver enzyme elevations [209,210]
Rilmenidine	Neurodegenerative disease	Inducer	mTOR/AMPK pathway	Enhances autophagy, improves disease progression in HD models [181]	Pilot clinical trial completed (EudraCT 2009-018119-14), further randomized trials planned	hypotension, dry mouth, dizziness [211]
Colchicine	Neurodegenerative disease	Inducer	Microtubules	Promotes autophagy by disrupting microtubule dynamics, aiding clearance of misfolded proteins [181]	Clinical trial ongoing (NCT03693781)	gastrointestinal upset, myelosuppression, neuropathy [212]
Tamoxifen	Neurodegenerative disease	Inducer	Estrogen receptor modulator	Enhances autophagy, reducing accumulation of misfolded proteins [181]	Clinical trial ongoing (NCT02166944)	hot flashes, thromboembolic events, endometrial cancer risk [213]
USP30 inhibitors	Neurodegenerative disease	Inducer	USP30	Promote mitophagy by inhibiting USP30, a negative regulator of mitochondrial clearance [182]	Entering Phase I trials	unknown, theoretical risks based on mechanism [182]
PINK1 activators	Neurodegenerative disease	Inducer	PINK1	Directly stimulates PINK1 to enhance selective mitophagy and improve mitochondrial quality [182]	Entering Phase I trials	unknown, theoretical risks based on mechanism [182]

## Abbreviations

The following abbreviations are used in this manuscript:

α-Syn	alpha-synuclein
Aβ	amyloid beta
AD	Alzheimer’s disease
Akt	protein kinase B
ALS	amyotrophic lateral sclerosis
AMBRA1	BECN1-regulated autophagy protein 1
AMPK	AMP-activated protein kinase
APP	amyloid precursor protein
ARIH1	ariadne RBR E3 ubiquitin protein ligase 1
ATF4	activating transcription factor 4
ATF5	activating transcription factor 5
ATP5A1	mitochondrial ATP synthase F(1) complex subunit alpha
ATPs	adenosine triphosphates
BNIP3	BCL2 interacting protein 3
C/EBP	CCAAT/enhancer-binding protein
CHOP	C/EBP homologous protein
ClpP	caseinolytic protease P
DELE1	DAP3-binding cell death enhancer 1
DNM1L	Dynamin-1-like protein
DRP1	Dynamin-related protein 1
eIF2α	eukaryotic translation initiation factor 2A
ER	endoplasmic reticulum
Erα	estrogen receptor alpha
Erk1/2	extracellular signal-regulated kinases 1 and 2
ETC	electron transport chain
FGF21	fibroblast growth factor 21
FOXO3A	transcription factor forkhead box protein O3a
FUNDC1	fun14 domain-containing protein 1
GABARAP	GABA type A receptor-associated protein
GAPDH	glyceraldehyde 3-phosphate dehydrogenase
GDF15	Growth differentiation factor 15
GP78	glycoprotein 78
HD	Huntington’s disease
HIF-1	hypoxia-inducible factor 1
HRI	Heme-regulated inhibitor kinase
HSP10	heat shock protein 10
HSP60	heat shock protein 60
HSP70	heat shock protein 70
HSP90	heat shock protein 90
HTT	wild-type huntingtin
IMS	intermembrane space
iPSCs	induced pluripotent stem cells
JNK	c-Jun N-terminal kinase
KRAS	oncogenic GTPase KRas
LC3B	light chain 3 beta
LIR	LC3 interaction region
Lonp1	Lon peptidase 1
Lys6	lysine 6
Lys11	lysine 11
Lys48	lysine 48
Lys63	lysine 63
MAP1A/MAP1B LC3	microtubule-associated protein 1A/1B-light chain 3
MAPKs	mitogen-activated protein kinases
Mdivi-1	mitochondrial division inhibitor 1
Mfn1	mitofusin 1
Mfn1/2	mitofusin 1/2
Mfn2	mitofusin 2
mHTT	mutant huntingtin
Mito-CP	mitochondria-targeted carboxy-proxyl
Mito-metformin	mitochondrial targeting of metformin
MQC	mitochondrial quality control
MRPP3	mitochondrial RNase P protein 3
MUL1	mitochondrial E3 ubiquitin protein ligase 1
mPTP	mitochondrial permeability transition pore
mRNA	messenger RNA
mTOR	mammalian (mechanistic) target of rapamycin
mtDNA	mitochondrial DNA
mtUPR	mitochondrial unfolded protein response
NAD^+^	nicotinamide adenine dinucleotide, oxidized form
NBR1	neighbor of BRCA1 gene 1
NDP52	calcium binding and coiled-coil domain 2
NIX	BNIP3-like protein X
NMJs	neuromuscular junctions
NMN	nicotinamide mononucleotide
NR	nicotinamide riboside
NRF1	nuclear respiratory factor 1
OPTN	optineurin
OPA1	mitochondrial Dynamin-like GTPase OPA1
OXPHOS	mitochondrial oxidative phosphorylation
p53	cellular tumor antigen p53
p62/SQSTM1	sequestosome 1
PD	Parkinson’s disease
PGC-1α	peroxisome proliferator-activated receptor gamma coactivator 1-alpha
PI3K	phosphoinositide-3-kinase
Pin1	peptidyl-prolyl cis-trans isomerase NIMA-interacting 1
PINK1	PTEN-induced putative kinase 1
polyQ	polyglutamine
PPP	pentose phosphate pathway
QC	quality control
Rab9a	ras-related protein Rab-9A
ROS	reactive oxygen species
rRNA	Ribosomal RNA
SIAH1	siah E3 ubiquitin protein ligase 1
SMURF1	SMAD-specific E3 ubiquitin protein ligase 1
SOD2	superoxide dismutase 2
SIRT1	sirtuin 1
SIRT3	sirtuin 3
STING	stimulator interferon genes
TAX1BP1	calcium binding and coiled-coil domain 3
TBK1	Tank-binding kinase 1
TDP-43	TAR DNA-binding protein 43
TFAM	mitochondrial transcription factor A
TIM	translocase of the inner membrane
TIM23	translocase of the inner membrane 23
TLR9	toll-like receptor 9
TOM	translocase of the outer membrane
TRAP1	Tumor necrosis factor receptor-associated protein 1
tRNA	Transfer RNA
Tyr18	tyrosine 18
Ub	ubiquitin
ULK1	Unc-51-like kinase 1
UCHL-1	ubiquitin C-terminal hydrolase L1
USP15	ubiquitin-specific protease 15
USP30	ubiquitin-specific protease 30
USP35	ubiquitin-specific protease 35
USP8	ubiquitin-specific protease 8
VCP	valosin-containing protein
VDAC1	voltage-dependent anion-selective channel 1

## References

[1] Hong W.-L., Huang H., Zeng X., Duan C.-Y. (2024). Targeting Mitochondrial Quality Control: New Therapeutic Strategies for Major Diseases. Mil. Med. Res..

[2] Smyrnias I. (2021). The Mitochondrial Unfolded Protein Response and Its Diverse Roles in Cellular Stress. Int. J. Biochem. Cell Biol..

[3] Ashton T.M., McKenna W.G., Kunz-Schughart L.A., Higgins G.S. (2018). Oxidative Phosphorylation as an Emerging Target in Cancer Therapy. Clin. Cancer Res..

[4] Zheng Y., Yang J., Li X., Qi L., Zheng Z., Kong J., Zhang G., Guo Y. (2025). Mitochondria at the Crossroads: Quality Control Mechanisms in Neuronal Senescence and Neurodegeneration. Neurobiol. Dis..

[5] Kenny T.C., Germain D. (2017). MtDNA, Metastasis, and the Mitochondrial Unfolded Protein Response (UPRmt). Front. Cell Dev. Biol..

[6] Yuan Y., Ju Y.S., Kim Y., Li J., Wang Y., Yoon C.J., Yang Y., Martincorena I., Creighton C.J., Weinstein J.N. (2020). Comprehensive Molecular Characterization of Mitochondrial Genomes in Human Cancers. Nat. Genet..

[7] Song J., Herrmann J.M., Becker T. (2021). Quality Control of the Mitochondrial Proteome. Nat. Rev. Mol. Cell Biol..

[8] Torres A.K., Fleischhart V., Inestrosa N.C. (2024). Mitochondrial Unfolded Protein Response (UPRmt): What We Know Thus Far. Front. Cell Dev. Biol..

[9] Zhang J., Qiao W., Luo Y. (2023). Mitochondrial Quality Control Proteases and Their Modulation for Cancer Therapy. Med. Res. Rev..

[10] Hertweck K.L., Dasgupta S. (2017). The Landscape of MtDNA Modifications in Cancer: A Tale of Two Cities. Front. Oncol..

[11] Rath S., Sharma R., Gupta R., Ast T., Chan C., Durham T.J., Goodman R.P., Grabarek Z., Haas M.E., Hung W.H.W. (2021). MitoCarta3.0: An Updated Mitochondrial Proteome Now with Sub-Organelle Localization and Pathway Annotations. Nucleic Acids Res..

[12] Chen Y., Zhang H., Zhou H., Ji W., Min W. (2016). Mitochondrial Redox Signaling and Tumor Progression. Cancers.

[13] Cilleros-Holgado P., Gómez-Fernández D., Piñero-Pérez R., Romero-Domínguez J.M., Reche-López D., López-Cabrera A., Álvarez-Córdoba M., Munuera-Cabeza M., Talaverón-Rey M., Suárez-Carrillo A. (2023). Mitochondrial Quality Control via Mitochondrial Unfolded Protein Response (MtUPR) in Ageing and Neurodegenerative Diseases. Biomolecules.

[14] Wang B., Chen W., Huang Q., Chen Y., Wang Y. (2024). Targeting Cancer Mitochondria by Inducing an Abnormal Mitochondrial Unfolded Protein Response Leads to Tumor Suppression. Int. J. Med. Sci..

[15] Ejma M., Madetko N., Brzecka A., Guranski K., Alster P., Misiuk-Hojło M., Somasundaram S.G., Kirkland C.E., Aliev G. (2020). The Links between Parkinson’s Disease and Cancer. Biomedicines.

[16] Basak B., Holzbaur E.L.F. (2025). Mitophagy in Neurons: Mechanisms Regulating Mitochondrial Turnover and Neuronal Homeostasis. J. Mol. Biol..

[17] Chacinska A., Koehler C.M., Milenkovic D., Lithgow T., Pfanner N. (2009). Importing Mitochondrial Proteins: Machineries and Mechanisms. Cell.

[18] Xia J., Jin J., Dai S., Fan H., Chen K., Li J., Luo F., Peng X. (2025). Mitophagy: A Key Regulator of Radiotherapy Resistance in the Tumor Immune Microenvironment. Mol. Asp. Med..

[19] Jain N., Chacinska A., Rehling P. (2025). Understanding Mitochondrial Protein Import: A Revised Model of the Presequence Translocase. Trends Biochem. Sci..

[20] Münch C. (2018). The Different Axes of the Mammalian Mitochondrial Unfolded Protein Response. BMC Biol..

[21] Kim Y.E., Hipp M.S., Bracher A., Hayer-Hartl M., Ulrich Hartl F. (2013). Molecular Chaperone Functions in Protein Folding and Proteostasis. Annu. Rev. Biochem..

[22] Okamoto T., Yamamoto H., Kudo I., Matsumoto K., Odaka M., Grave E., Itoh H. (2017). HSP60 Possesses a GTPase Activity and Mediates Protein Folding with HSP10. Sci. Rep..

[23] Walter S., Buchner J. (2002). Molecular Chaperones—Cellular Machines for Protein Folding. Angew. Chem. Int. Ed..

[24] Song H.Y., Dunbar J.D., Zhang Y.X., Guo D., Donner D.B. (1995). Identification of a Protein with Homology to Hsp90 That Binds the Type 1 Tumor Necrosis Factor Receptor. J. Biol. Chem..

[25] Gesualdi N.M., Chirico G., Pirozzi G., Costantino E., Landriscina M., Esposito F. (2007). Tumor Necrosis Factor-Associated Protein 1 (TRAP-1) Protects Cells from Oxidative Stress and Apoptosis. Stress.

[26] Webb C.T., Gorman M.A., Lazarou M., Ryan M.T., Gulbis J.M. (2006). Crystal Structure of the Mitochondrial Chaperone TIM9•10 Reveals a Six-Bladed α-Propeller. Mol. Cell.

[27] Haroon S., Vermulst M. (2016). Linking Mitochondrial Dynamics to Mitochondrial Protein Quality Control. Curr. Opin. Genet. Dev..

[28] Naresh N.U., Haynes C.M. (2019). Signaling and Regulation of the Mitochondrial Unfolded Protein Response. Cold Spring Harb. Perspect. Biol..

[29] Qureshi M.A., Haynes C.M., Pellegrino M.W. (2017). The Mitochondrial Unfolded Protein Response: Signaling from the Powerhouse. J. Biol. Chem..

[30] Haynes C.M., Ron D. (2010). The Mitochondrial UPR—Protecting Organelle Protein Homeostasis. J. Cell Sci..

[31] Shpilka T., Du Y., Yang Q., Melber A., Uma Naresh N., Lavelle J., Kim S., Liu P., Weidberg H., Li R. (2021). UPRmt Scales Mitochondrial Network Expansion with Protein Synthesis via Mitochondrial Import in Caenorhabditis Elegans. Nat. Commun..

[32] Rolland S.G., Schneid S., Schwarz M., Rackles E., Fischer C., Haeussler S., Regmi S.G., Yeroslaviz A., Habermann B., Mokranjac D. (2019). Compromised Mitochondrial Protein Import Acts as a Signal for UPRmt. Cell Rep..

[33] Guo Z., Tian Y., Gao J., Zhou B., Zhou X., Chang X., Zhou H. (2024). Enhancement of Mitochondrial Homeostasis: A Novel Approach to Attenuate Hypoxic Myocardial Injury. Int. J. Med. Sci..

[34] Münch C., Harper J.W. (2016). Mitochondrial Unfolded Protein Response Controls Matrix Pre-RNA Processing and Translation. Nature.

[35] Papa L., Germain D. (2011). Estrogen Receptor Mediates a Distinct Mitochondrial Unfolded Protein Response. J. Cell Sci..

[36] Skorko-Glonek J., Zurawa-Janicka D., Koper T., Jarzab M., Figaj D., Glaza P., Lipinska B. (2012). HtrA Protease Family as Therapeutic Targets. Curr. Pharm. Des..

[37] Zhang J., Xiang H., Liu J., Chen Y., He R.-R., Liu B. (2020). Mitochondrial Sirtuin 3: New Emerging Biological Function and Therapeutic Target. Theranostics.

[38] Tseng A.H.H., Shieh S.-S., Wang D.L. (2013). SIRT3 Deacetylates FOXO3 to Protect Mitochondria against Oxidative Damage. Free Radic. Biol. Med..

[39] Lin Y.-F., Schulz A.M., Pellegrino M.W., Lu Y., Shaham S., Haynes C.M. (2016). Maintenance and Propagation of a Deleterious Mitochondrial Genome by the Mitochondrial Unfolded Protein Response. Nature.

[40] Kim I., Rodriguez-Enriquez S., Lemasters J.J. (2007). Selective Degradation of Mitochondria by Mitophagy. Arch. Biochem. Biophys..

[41] Lemasters J.J. (2005). Selective Mitochondrial Autophagy, or Mitophagy, as a Targeted Defense Against Oxidative Stress, Mitochondrial Dysfunction, and Aging. Rejuvenation Res..

[42] Palikaras K., Lionaki E., Tavernarakis N. (2018). Mechanisms of Mitophagy in Cellular Homeostasis, Physiology and Pathology. Nat. Cell Biol..

[43] Palikaras K., Princz A., Tavernarakis N. (2018). Mitophagy Modulators. Encyclopedia of Biomedical Gerontology.

[44] Gersch M., Gladkova C., Schubert A.F., Michel M.A., Maslen S., Komander D. (2017). Mechanism and Regulation of the Lys6-Selective Deubiquitinase USP30. Nat. Struct. Mol. Biol..

[45] Bingol B., Sheng M. (2016). Mechanisms of Mitophagy: PINK1, Parkin, USP30 and Beyond. Free Radic. Biol. Med..

[46] Durcan T.M., Tang M.Y., Pérusse J.R., Dashti E.A., Aguileta M.A., McLelland G., Gros P., Shaler T.A., Faubert D., Coulombe B. (2014). USP8 Regulates Mitophagy by Removing K6-linked Ubiquitin Conjugates from Parkin. EMBO J..

[47] Bingol B., Tea J.S., Phu L., Reichelt M., Bakalarski C.E., Song Q., Foreman O., Kirkpatrick D.S., Sheng M. (2014). The Mitochondrial Deubiquitinase USP30 Opposes Parkin-Mediated Mitophagy. Nature.

[48] Villa E., Marchetti S., Ricci J.-E. (2018). No Parkin Zone: Mitophagy without Parkin. Trends Cell Biol..

[49] Onishi M., Yamano K., Sato M., Matsuda N., Okamoto K. (2021). Molecular Mechanisms and Physiological Functions of Mitophagy. EMBO J..

[50] Wang X.-L., Feng S.-T., Wang Z.-Z., Chen N.-H., Zhang Y. (2021). Role of Mitophagy in Mitochondrial Quality Control: Mechanisms and Potential Implications for Neurodegenerative Diseases. Pharmacol. Res..

[51] Zheng X., Boyer L., Jin M., Mertens J., Kim Y., Ma L., Ma L., Hamm M., Gage F.H., Hunter T. (2016). Metabolic Reprogramming during Neuronal Differentiation from Aerobic Glycolysis to Neuronal Oxidative Phosphorylation. eLife.

[52] Bolaños J.P., Almeida A. (2010). The Pentose-phosphate Pathway in Neuronal Survival against Nitrosative Stress. IUBMB Life.

[53] Quintana D.D., Garcia J.A., Anantula Y., Rellick S.L., Engler-Chiurazzi E.B., Sarkar S.N., Brown C.M., Simpkins J.W. (2020). Amyloid-β Causes Mitochondrial Dysfunction via a Ca^2+^-Driven Upregulation of Oxidative Phosphorylation and Superoxide Production in Cerebrovascular Endothelial Cells. J. Alzheimer’s Dis..

[54] Keeney P.M., Xie J., Capaldi R.A., Bennett J.P. (2006). Parkinson’s Disease Brain Mitochondrial Complex I Has Oxidatively Damaged Subunits and Is Functionally Impaired and Misassembled. J. Neurosci..

[55] Lill C.M. (2016). Genetics of Parkinson’s Disease. Mol. Cell Probes.

[56] Wilkaniec A., Strosznajder J.B., Adamczyk A. (2013). Toxicity of Extracellular Secreted Alpha-Synuclein: Its Role in Nitrosative Stress and Neurodegeneration. Neurochem. Int..

[57] Ganjam G.K., Bolte K., Matschke L.A., Neitemeier S., Dolga A.M., Höllerhage M., Höglinger G.U., Adamczyk A., Decher N., Oertel W.H. (2019). Mitochondrial Damage by α-Synuclein Causes Cell Death in Human Dopaminergic Neurons. Cell Death Dis..

[58] Pozo Devoto V.M., Dimopoulos N., Alloatti M., Pardi M.B., Saez T.M., Otero M.G., Cromberg L.E., Marín-Burgin A., Scassa M.E., Stokin G.B. (2017). ASynuclein Control of Mitochondrial Homeostasis in Human-Derived Neurons Is Disrupted by Mutations Associated with Parkinson’s Disease. Sci. Rep..

[59] Wilkaniec A., Cieślik M., Murawska E., Babiec L., Gąssowska-Dobrowolska M., Pałasz E., Jęśko H., Adamczyk A. (2020). P2X7 Receptor Is Involved in Mitochondrial Dysfunction Induced by Extracellular Alpha Synuclein in Neuroblastoma SH-SY5Y Cells. Int. J. Mol. Sci..

[60] Joshi D.C., Chavan M.B., Gurow K., Gupta M., Dhaliwal J.S., Ming L.C. (2025). The Role of Mitochondrial Dysfunction in Huntington’s Disease: Implications for Therapeutic Targeting. Biomed. Pharmacother..

[61] Bossy-Wetzel E., Petrilli A., Knott A.B. (2008). Mutant Huntingtin and Mitochondrial Dysfunction. Trends Neurosci..

[62] Bannwarth S., Ait-El-Mkadem S., Chaussenot A., Genin E.C., Lacas-Gervais S., Fragaki K., Berg-Alonso L., Kageyama Y., Serre V., Moore D.G. (2014). A Mitochondrial Origin for Frontotemporal Dementia and Amyotrophic Lateral Sclerosis through CHCHD10 Involvement. Brain.

[63] Genin E.C., Plutino M., Bannwarth S., Villa E., Cisneros-Barroso E., Roy M., Ortega-Vila B., Fragaki K., Lespinasse F., Pinero-Martos E. (2016). *CHCHD10* Mutations Promote Loss of Mitochondrial Cristae Junctions with Impaired Mitochondrial Genome Maintenance and Inhibition of Apoptosis. EMBO Mol. Med..

[64] Brown C.A., Lally C., Kupelian V., Flanders W.D. (2021). Estimated Prevalence and Incidence of Amyotrophic Lateral Sclerosis and SOD1 and C9orf72 Genetic Variants. Neuroepidemiology.

[65] Choi S.Y., Lopez-Gonzalez R., Krishnan G., Phillips H.L., Li A.N., Seeley W.W., Yao W.-D., Almeida S., Gao F.-B. (2019). C9ORF72-ALS/FTD-Associated Poly(GR) Binds Atp5a1 and Compromises Mitochondrial Function In Vivo. Nat. Neurosci..

[66] Jordan J., de Groot P.W.J., Galindo M.F. (2011). Mitochondria: The Headquarters in Ischemia-Induced Neuronal Death. Cent. Nerv. Syst. Agents Med. Chem..

[67] Tao G., Liao W., Hou J., Jiang X., Deng X., Chen G., Ding C. (2024). Advances in Crosstalk among Innate Immune Pathways Activated by Mitochondrial DNA. Heliyon.

[68] Cheng J., Zhang R., Xu Z., Ke Y., Sun R., Yang H., Zhang X., Zhen X., Zheng L.-T. (2021). Early Glycolytic Reprogramming Controls Microglial Inflammatory Activation. J. Neuroinflamm..

[69] Ji T., Zhang X., Xin Z., Xu B., Jin Z., Wu J., Hu W., Yang Y. (2020). Does Perturbation in the Mitochondrial Protein Folding Pave the Way for Neurodegeneration Diseases?. Ageing Res. Rev..

[70] Beck J.S., Mufson E.J., Counts S.E. (2016). Evidence for Mitochondrial UPR Gene Activation in Familial and Sporadic Alzheimer’s Disease. Curr. Alzheimer Res..

[71] Counts S.E., Kelly S.C., Weinberg R.B., Beck J.S. (2017). [P2–179]: Mitochondrial Unfolded Protein Response (mtUPR) Dysfunction During the Progression of Alzheimer’s Disease. Alzheimer’s Dement..

[72] Wang W., Ma X., Bhatta S., Shao C., Zhao F., Fujioka H., Torres S., Wu F., Zhu X. (2023). Intraneuronal β-Amyloid Impaired Mitochondrial Proteostasis through the Impact on LONP1. Proc. Natl. Acad. Sci. USA.

[73] de Castro I.P., Martins L.M., Loh S.H.Y. (2011). Mitochondrial Quality Control and Parkinson’s Disease: A Pathway Unfolds. Mol. Neurobiol..

[74] Pimenta de Castro I., Costa A.C., Lam D., Tufi R., Fedele V., Moisoi N., Dinsdale D., Deas E., Loh S.H.Y., Martins L.M. (2012). Genetic Analysis of Mitochondrial Protein Misfolding in Drosophila Melanogaster. Cell Death Differ..

[75] Martinez B.A., Petersen D.A., Gaeta A.L., Stanley S.P., Caldwell G.A., Caldwell K.A. (2017). Dysregulation of the Mitochondrial Unfolded Protein Response Induces Non-Apoptotic Dopaminergic Neurodegeneration in *C. elegans* Models of Parkinson’s Disease. J. Neurosci..

[76] Almeida L.M., Oliveira Â., Oliveira J.M.A., Pinho B.R. (2023). Stress Response Mechanisms in Protein Misfolding Diseases: Profiling a Cellular Model of Huntington’s Disease. Arch. Biochem. Biophys..

[77] Fu Z., Liu F., Liu C., Jin B., Jiang Y., Tang M., Qi X., Guo X. (2019). Mutant Huntingtin Inhibits the Mitochondrial Unfolded Protein Response by Impairing ABCB10 MRNA Stability. Biochim. Biophys. Acta (BBA)—Mol. Basis Dis..

[78] Wang P., Deng J., Dong J., Liu J., Bigio E.H., Mesulam M., Wang T., Sun L., Wang L., Lee A.Y.-L. (2019). TDP-43 Induces Mitochondrial Damage and Activates the Mitochondrial Unfolded Protein Response. PLoS Genet..

[79] Narendra D., Tanaka A., Suen D.-F., Youle R.J. (2008). Parkin Is Recruited Selectively to Impaired Mitochondria and Promotes Their Autophagy. J. Cell Biol..

[80] Burman J.L., Yu S., Poole A.C., Decal R.B., Pallanck L. (2012). Analysis of Neural Subtypes Reveals Selective Mitochondrial Dysfunction in Dopaminergic Neurons from *Parkin* Mutants. Proc. Natl. Acad. Sci. USA.

[81] Choubey V., Safiulina D., Vaarmann A., Cagalinec M., Wareski P., Kuum M., Zharkovsky A., Kaasik A. (2011). Mutant A53T α-Synuclein Induces Neuronal Death by Increasing Mitochondrial Autophagy. J. Biol. Chem..

[82] Chinta S.J., Mallajosyula J.K., Rane A., Andersen J.K. (2010). Mitochondrial Alpha-Synuclein Accumulation Impairs Complex I Function in Dopaminergic Neurons and Results in Increased Mitophagy In Vivo. Neurosci. Lett..

[83] Shaltouki A., Hsieh C.-H., Kim M.J., Wang X. (2018). Alpha-Synuclein Delays Mitophagy and Targeting Miro Rescues Neuron Loss in Parkinson’s Models. Acta Neuropathol..

[84] Wilkaniec A., Lenkiewicz A.M., Czapski G.A., Jęśko H.M., Hilgier W., Brodzik R., Gąssowska-Dobrowolska M., Culmsee C., Adamczyk A. (2019). Extracellular Alpha-Synuclein Oligomers Induce Parkin S-Nitrosylation: Relevance to Sporadic Parkinson’s Disease Etiopathology. Mol. Neurobiol..

[85] Wilkaniec A., Lenkiewicz A.M., Babiec L., Murawska E., Jęśko H.M., Cieślik M., Culmsee C., Adamczyk A. (2021). Exogenous Alpha-Synuclein Evoked Parkin Downregulation Promotes Mitochondrial Dysfunction in Neuronal Cells. Implic. Park. Dis. Pathology. Front. Aging Neurosci..

[86] Fang E.F., Hou Y., Palikaras K., Adriaanse B.A., Kerr J.S., Yang B., Lautrup S., Hasan-Olive M.M., Caponio D., Dan X. (2019). Mitophagy Inhibits Amyloid-β and Tau Pathology and Reverses Cognitive Deficits in Models of Alzheimer’s Disease. Nat. Neurosci..

[87] Moreira P.I., Siedlak S.L., Wang X., Santos M.S., Oliveira C.R., Tabaton M., Nunomura A., Szweda L.I., Aliev G., Smith M.A. (2007). Increased Autophagic Degradation of Mitochondria in Alzheimer Disease. Autophagy.

[88] Martín-Maestro P., Gargini R., Perry G., Avila J., García-Escudero V. (2016). PARK2 Enhancement Is Able to Compensate Mitophagy Alterations Found in Sporadic Alzheimer’s Disease. Hum. Mol. Genet..

[89] Ye X., Sun X., Starovoytov V., Cai Q. (2015). Parkin-Mediated Mitophagy in Mutant HAPP Neurons and Alzheimer’s Disease Patient Brains. Hum. Mol. Genet..

[90] Tammineni P., Jeong Y.Y., Feng T., Aikal D., Cai Q. (2017). Impaired Axonal Retrograde Trafficking of the Retromer Complex Augments Lysosomal Deficits in Alzheimer’s Disease Neurons. Hum. Mol. Genet..

[91] Corsetti V., Florenzano F., Atlante A., Bobba A., Ciotti M.T., Natale F., Della Valle F., Borreca A., Manca A., Meli G. (2015). NH2-Truncated Human Tau Induces Deregulated Mitophagy in Neurons by Aberrant Recruitment of Parkin and UCHL-1: Implications in Alzheimer’s Disease. Hum. Mol. Genet..

[92] Cummins N., Tweedie A., Zuryn S., Bertran-Gonzalez J., Götz J. (2019). Disease-associated Tau Impairs Mitophagy by Inhibiting Parkin Translocation to Mitochondria. EMBO J..

[93] Tak Y.J., Park J.-H., Rhim H., Kang S. (2020). ALS-Related Mutant SOD1 Aggregates Interfere with Mitophagy by Sequestering the Autophagy Receptor Optineurin. Int. J. Mol. Sci..

[94] Rogers R.S., Tungtur S., Tanaka T., Nadeau L.L., Badawi Y., Wang H., Ni H.-M., Ding W.-X., Nishimune H. (2017). Impaired Mitophagy Plays a Role in Denervation of Neuromuscular Junctions in ALS Mice. Front. Neurosci..

[95] de Calbiac H., Renault S., Haouy G., Jung V., Roger K., Zhou Q., Campanari M.-L., Chentout L., Demy D.L., Marian A. (2024). Poly-GP Accumulation Due to C9orf72 Loss of Function Induces Motor Neuron Apoptosis through Autophagy and Mitophagy Defects. Autophagy.

[96] Perera N.D., Sheean R.K., Lau C.L., Shin Y.S., Beart P.M., Horne M.K., Turner B.J. (2018). Rilmenidine Promotes MTOR-Independent Autophagy in the Mutant SOD1 Mouse Model of Amyotrophic Lateral Sclerosis without Slowing Disease Progression. Autophagy.

[97] Franco-Iborra S., Plaza-Zabala A., Montpeyo M., Sebastian D., Vila M., Martinez-Vicente M. (2021). Mutant HTT (Huntingtin) Impairs Mitophagy in a Cellular Model of Huntington Disease. Autophagy.

[98] Hwang S., Disatnik M., Mochly-Rosen D. (2015). Impaired GAPDH-induced Mitophagy Contributes to the Pathology of Huntington’s Disease. EMBO Mol. Med..

[99] Guo X., Sun X., Hu D., Wang Y.-J., Fujioka H., Vyas R., Chakrapani S., Joshi A.U., Luo Y., Mochly-Rosen D. (2016). VCP Recruitment to Mitochondria Causes Mitophagy Impairment and Neurodegeneration in Models of Huntington’s Disease. Nat. Commun..

[100] Choi W., Fattah M., Shang Y., Thompson M.P., Carrow K.P., Hu D., Liu Z., Avram M.J., Bailey K., Berger O. (2024). Proteomimetic Polymer Blocks Mitochondrial Damage, Rescues Huntington’s Neurons, and Slows Onset of Neuropathology in Vivo. Sci. Adv..

[101] Khalil B., El Fissi N., Aouane A., Cabirol-Pol M.-J., Rival T., Liévens J.-C. (2015). PINK1-Induced Mitophagy Promotes Neuroprotection in Huntington’s Disease. Cell Death Dis..

[102] Beatriz M., Vilaça R., Anjo S.I., Manadas B., Januário C., Rego A.C., Lopes C. (2022). Defective Mitochondria-lysosomal Axis Enhances the Release of Extracellular Vesicles Containing Mitochondrial DNA and Proteins in Huntington’s Disease. J. Extracell. Biol..

[103] Kenny T.C., Craig A.J., Villanueva A., Germain D. (2019). Mitohormesis Primes Tumor Invasion and Metastasis. Cell Rep..

[104] Zimmers T.A., Jin X., Gutierrez J.C., Acosta C., McKillop I.H., Pierce R.H., Koniaris L.G. (2008). Effect of in Vivo Loss of GDF-15 on Hepatocellular Carcinogenesis. J. Cancer Res. Clin. Oncol..

[105] Lee J.J., van de Ven R.A.H., Zaganjor E., Ng M.R., Barakat A., Demmers J.J.P.G., Finley L.W.S., Gonzalez Herrera K.N., Hung Y.P., Harris I.S. (2018). Inhibition of Epithelial Cell Migration and Src/FAK Signaling by SIRT3. Proc. Natl. Acad. Sci. USA.

[106] Huang S., Chen X., Zheng J., Huang Y., Song L., Yin Y., Xiong J. (2017). Low SIRT3 Expression Contributes to Tumor Progression, Development and Poor Prognosis in Human Pancreatic Carcinoma. Pathol. Res. Pract..

[107] Finley L.W.S., Carracedo A., Lee J., Souza A., Egia A., Zhang J., Teruya-Feldstein J., Moreira P.I., Cardoso S.M., Clish C.B. (2011). SIRT3 Opposes Reprogramming of Cancer Cell Metabolism through HIF1α Destabilization. Cancer Cell.

[108] Castilla C., Congregado B., Conde J.M., Medina R., Torrubia F.J., Japón M.A., Sáez C. (2010). Immunohistochemical Expression of Hsp60 Correlates with Tumor Progression and Hormone Resistance in Prostate Cancer. Urology.

[109] Hamelin C., Cornut E., Poirier F., Pons S., Beaulieu C., Charrier J., Haïdous H., Cotte E., Lambert C., Piard F. (2011). Identification and Verification of Heat Shock Protein 60 as a Potential Serum Marker for Colorectal Cancer. FEBS J..

[110] Na Y., Kaul S.C., Ryu J., Lee J.-S., Ahn H.M., Kaul Z., Kalra R.S., Li L., Widodo N., Yun C.-O. (2016). Stress Chaperone Mortalin Contributes to Epithelial-to-Mesenchymal Transition and Cancer Metastasis. Cancer Res..

[111] Kang B.H., Plescia J., Dohi T., Rosa J., Doxsey S.J., Altieri D.C. (2007). Regulation of Tumor Cell Mitochondrial Homeostasis by an Organelle-Specific Hsp90 Chaperone Network. Cell.

[112] Siegelin M.D., Dohi T., Raskett C.M., Orlowski G.M., Powers C.M., Gilbert C.A., Ross A.H., Plescia J., Altieri D.C. (2011). Exploiting the Mitochondrial Unfolded Protein Response for Cancer Therapy in Mice and Human Cells. J. Clin. Investig..

[113] Ghosh J.C., Dohi T., Kang B.H., Altieri D.C. (2008). Hsp60 Regulation of Tumor Cell Apoptosis. J. Biol. Chem..

[114] Pavlides S., Whitaker-Menezes D., Castello-Cros R., Flomenberg N., Witkiewicz A.K., Frank P.G., Casimiro M.C., Wang C., Fortina P., Addya S. (2009). The Reverse Warburg Effect: Aerobic Glycolysis in Cancer Associated Fibroblasts and the Tumor Stroma. Cell Cycle.

[115] Gibellini L., Pinti M., Boraldi F., Giorgio V., Bernardi P., Bartolomeo R., Nasi M., De Biasi S., Missiroli S., Carnevale G. (2014). Silencing of Mitochondrial Lon Protease Deeply Impairs Mitochondrial Proteome and Function in Colon Cancer Cells. FASEB J..

[116] Di K., Lomeli N., Wood S.D., Vanderwal C.D., Bota D.A. (2016). Mitochondrial Lon Is Over-Expressed in High-Grade Gliomas, and Mediates Hypoxic Adaptation: Potential Role of Lon as a Therapeutic Target in Glioma. Oncotarget.

[117] Seo J.H., Rivadeneira D.B., Caino M.C., Chae Y.C., Speicher D.W., Tang H.-Y., Vaira V., Bosari S., Palleschi A., Rampini P. (2016). The Mitochondrial Unfoldase-Peptidase Complex ClpXP Controls Bioenergetics Stress and Metastasis. PLoS Biol..

[118] Cole A., Wang Z., Coyaud E., Voisin V., Gronda M., Jitkova Y., Mattson R., Hurren R., Babovic S., Maclean N. (2015). Inhibition of the Mitochondrial Protease ClpP as a Therapeutic Strategy for Human Acute Myeloid Leukemia. Cancer Cell.

[119] Monaco S.E., Angelastro J.M., Szabolcs M., Greene L.A. (2007). The Transcription Factor ATF5 Is Widely Expressed in Carcinomas, and Interference with Its Function Selectively Kills Neoplastic, but Not Nontransformed, Breast Cell Lines. Int. J. Cancer.

[120] Chen A., Qian D., Wang B., Hu M., Lu J., Qi Y., Liu D.X. (2012). ATF5 Is Overexpressed in Epithelial Ovarian Carcinomas and Interference with Its Function Increases Apoptosis Through the Downregulation of Bcl-2 in SKOV-3 Cells. Int. J. Gynecol. Pathol..

[121] Li G., Xu Y., Guan D., Liu Z., Liu D.X. (2011). HSP70 Protein Promotes Survival of C6 and U87 Glioma Cells by Inhibition of ATF5 Degradation. J. Biol. Chem..

[122] Nukuda A., Endoh H., Yasuda M., Mizutani T., Kawabata K., Haga H. (2016). Role of ATF5 in the Invasive Potential of Diverse Human Cancer Cell Lines. Biochem. Biophys. Res. Commun..

[123] Hu M., Wang B., Qian D., Li L., Zhang L., Song X., Liu D.X. (2012). Interference with ATF5 Function Enhances the Sensitivity of Human Pancreatic Cancer Cells to Paclitaxel-Induced Apoptosis. Anticancer Res..

[124] Yan C., Li T. (2018). Dual Role of Mitophagy in Cancer Drug Resistance. Anticancer Res..

[125] Wang Y., Liu H.-H., Cao Y.-T., Zhang L.-L., Huang F., Yi C. (2020). The Role of Mitochondrial Dynamics and Mitophagy in Carcinogenesis, Metastasis and Therapy. Front. Cell Dev. Biol..

[126] Guo W., Sun Y., Liu W., Wu X., Guo L., Cai P., Wu X., Wu X., Shen Y., Shu Y. (2014). Small Molecule-Driven Mitophagy-Mediated NLRP3 Inflammasome Inhibition Is Responsible for the Prevention of Colitis-Associated Cancer. Autophagy.

[127] Xu H.-M., Hu F. (2022). The Role of Autophagy and Mitophagy in Cancers. Arch. Physiol. Biochem..

[128] Chang C.-H., Qiu J., O’Sullivan D., Buck M.D., Noguchi T., Curtis J.D., Chen Q., Gindin M., Gubin M.M., van der Windt G.J.W. (2015). Metabolic Competition in the Tumor Microenvironment Is a Driver of Cancer Progression. Cell.

[129] Apel A., Herr I., Schwarz H., Rodemann H.P., Mayer A. (2008). Blocked Autophagy Sensitizes Resistant Carcinoma Cells to Radiation Therapy. Cancer Res..

[130] Zheng R., Yao Q., Xie G., Du S., Ren C., Wang Y., Yuan Y. (2015). TAT-ODD-P53 Enhances the Radiosensitivity of Hypoxic Breast Cancer Cells by Inhibiting Parkin-Mediated Mitophagy. Oncotarget.

[131] Liu J., Chen Z., Guo J., Wang L., Liu X. (2019). Ambra1 Induces Autophagy and Desensitizes Human Prostate Cancer Cells to Cisplatin. Biosci. Rep..

[132] Hou H., Er P., Cheng J., Chen X., Ding X., Wang Y., Chen X., Yuan Z., Pang Q., Wang P. (2017). High Expression of FUNDC1 Predicts Poor Prognostic Outcomes and Is a Promising Target to Improve Chemoradiotherapy Effects in Patients with Cervical Cancer. Cancer Med..

[133] MacKeigan J.P., Murphy L.O., Blenis J. (2005). Sensitized RNAi Screen of Human Kinases and Phosphatases Identifies New Regulators of Apoptosis and Chemoresistance. Nat. Cell Biol..

[134] Dany M., Gencer S., Nganga R., Thomas R.J., Oleinik N., Baron K.D., Szulc Z.M., Ruvolo P., Kornblau S., Andreeff M. (2016). Targeting FLT3-ITD Signaling Mediates Ceramide-Dependent Mitophagy and Attenuates Drug Resistance in AML. Blood.

[135] Fujiwara M., Marusawa H., Wang H.-Q., Iwai A., Ikeuchi K., Imai Y., Kataoka A., Nukina N., Takahashi R., Chiba T. (2008). Parkin as a Tumor Suppressor Gene for Hepatocellular Carcinoma. Oncogene.

[136] Matsuda S. (2015). Functions and Characteristics of PINK1 and Parkin in Cancer. Front. Biosci..

[137] D’Amico A.G., Maugeri G., Magro G., Salvatorelli L., Drago F., D’Agata V. (2015). Expression Pattern of Parkin Isoforms in Lung Adenocarcinomas. Tumor Biol..

[138] Tay S.-P., Yeo C.W.S., Chai C., Chua P.-J., Tan H.-M., Ang A.X.Y., Yip D.L.H., Sung J.-X., Tan P.H., Bay B.-H. (2010). Parkin Enhances the Expression of Cyclin-Dependent Kinase 6 and Negatively Regulates the Proliferation of Breast Cancer Cells. J. Biol. Chem..

[139] Maugeri G., D’Amico A.G., Magro G., Salvatorelli L., Barbagallo G.M.V., Saccone S., Drago F., Cavallaro S., D’Agata V. (2015). Expression Profile of Parkin Isoforms in Human Gliomas. Int. J. Oncol..

[140] Hu H.-H., Kannengiesser C., Lesage S., André J., Mourah S., Michel L., Descamps V., Basset-Seguin N., Bagot M., Bensussan A. (2016). *PARKIN* Inactivation Links Parkinson’s Disease to Melanoma. J. Natl. Cancer Inst..

[141] Poulogiannis G., McIntyre R.E., Dimitriadi M., Apps J.R., Wilson C.H., Ichimura K., Luo F., Cantley L.C., Wyllie A.H., Adams D.J. (2010). *PARK2* Deletions Occur Frequently in Sporadic Colorectal Cancer and Accelerate Adenoma Development in *Apc* Mutant Mice. Proc. Natl. Acad. Sci. USA.

[142] Veeriah S., Taylor B.S., Meng S., Fang F., Yilmaz E., Vivanco I., Janakiraman M., Schultz N., Hanrahan A.J., Pao W. (2010). Somatic Mutations of the Parkinson’s Disease–Associated Gene PARK2 in Glioblastoma and Other Human Malignancies. Nat. Genet..

[143] Lee Y.S., Jung Y.Y., Park M.H., Yeo I.J., Im H.S., Nam K.T., Kim H.D., Kang S.K., Song J.K., Kim Y.R. (2018). Deficiency of Parkin Suppresses Melanoma Tumor Development and Metastasis through Inhibition of MFN2 Ubiquitination. Cancer Lett..

[144] Sun X., Liu M., Hao J., Li D., Luo Y., Wang X., Yang Y., Li F., Shui W., Chen Q. (2013). Parkin Deficiency Contributes to Pancreatic Tumorigenesis by Inducing Spindle Multipolarity and Misorientation. Cell Cycle.

[145] Agnihotri S., Golbourn B., Huang X., Remke M., Younger S., Cairns R.A., Chalil A., Smith C.A., Krumholtz S.-L., Mackenzie D. (2016). PINK1 Is a Negative Regulator of Growth and the Warburg Effect in Glioblastoma. Cancer Res..

[146] Liu L., Zuo Z., Lu S., Wang L., Liu A., Liu X. (2018). Silencing of PINK1 Represses Cell Growth, Migration and Induces Apoptosis of Lung Cancer Cells. Biomed. Pharmacother..

[147] Yamashita K., Miyata H., Makino T., Masuike Y., Furukawa H., Tanaka K., Miyazaki Y., Takahashi T., Kurokawa Y., Yamasaki M. (2017). High Expression of the Mitophagy-Related Protein Pink1 Is Associated with a Poor Response to Chemotherapy and a Poor Prognosis for Patients Treated with Neoadjuvant Chemotherapy for Esophageal Squamous Cell Carcinoma. Ann. Surg. Oncol..

[148] Chourasia A.H., Tracy K., Frankenberger C., Boland M.L., Sharifi M.N., Drake L.E., Sachleben J.R., Asara J.M., Locasale J.W., Karczmar G.S. (2015). Mitophagy Defects Arising from BNip3 Loss Promote Mammary Tumor Progression to Metastasis. EMBO Rep..

[149] Erkan M., Kleeff J., Esposito I., Giese T., Ketterer K., Büchler M.W., Giese N.A., Friess H. (2005). Loss of BNIP3 Expression Is a Late Event in Pancreatic Cancer Contributing to Chemoresistance and Worsened Prognosis. Oncogene.

[150] Okami J., Simeone D.M., Logsdon C.D. (2004). Silencing of the Hypoxia-Inducible Cell Death Protein BNIP3 in Pancreatic Cancer. Cancer Res..

[151] Chourasia A.H., Boland M.L., Macleod K.F. (2015). Mitophagy and Cancer. Cancer Metab..

[152] Sowter H.M., Ferguson M., Pym C., Watson P., Fox S.B., Han C., Harris A.L. (2003). Expression of the Cell Death Genes BNip3 and NIX in Ductal Carcinoma in Situ of the Breast; Correlation of BNip3 Levels with Necrosis and Grade. J. Pathol..

[153] Humpton T.J., Alagesan B., DeNicola G.M., Lu D., Yordanov G.N., Leonhardt C.S., Yao M.A., Alagesan P., Zaatari M.N., Park Y. (2019). Oncogenic KRAS Induces NIX-Mediated Mitophagy to Promote Pancreatic Cancer. Cancer Discov..

[154] Li W., Li Y., Siraj S., Jin H., Fan Y., Yang X., Huang X., Wang X., Wang J., Liu L. (2019). FUN14 Domain-Containing 1–Mediated Mitophagy Suppresses Hepatocarcinogenesis by Inhibition of Inflammasome Activation in Mice. Hepatology.

[155] Wang G., Fan Y., Cao P., Tan K. (2022). Insight into the Mitochondrial Unfolded Protein Response and Cancer: Opportunities and Challenges. Cell Biosci..

[156] Macdonald R., Barnes K., Hastings C., Mortiboys H. (2018). Mitochondrial Abnormalities in Parkinson’s Disease and Alzheimer’s Disease: Can. Mitochondria Be Targeted Therapeutically?. Biochem. Soc. Trans..

[157] Houck A.L., Seddighi S., Driver J.A. (2019). At the Crossroads Between Neurodegeneration and Cancer: A Review of Overlapping Biology and Its Implications. Curr. Aging Sci..

[158] Swerdlow R.H. (2018). Mitochondria and Mitochondrial Cascades in Alzheimer’s Disease. J. Alzheimer’s Dis..

[159] Urra F.A., Muñoz F., Lovy A., Cárdenas C. (2017). The Mitochondrial Complex(I)Ty of Cancer. Front. Oncol..

[160] Warburg O. (1956). On the Origin of Cancer Cells. Science (1979).

[161] O’Callaghan B., Hardy J., Plun-Favreau H. (2023). PINK1: From Parkinson’s Disease to Mitophagy and Back Again. PLoS Biol..

[162] Sorrentino V., Romani M., Mouchiroud L., Beck J.S., Zhang H., D’Amico D., Moullan N., Potenza F., Schmid A.W., Rietsch S. (2017). Enhancing Mitochondrial Proteostasis Reduces Amyloid-β Proteotoxicity. Nature.

[163] Driver J.A. (2014). Inverse Association between Cancer and Neurodegenerative Disease: Review of the Epidemiologic and Biological Evidence. Biogerontology.

[164] Brasnjevic I., Hof P.R., Steinbusch H.W.M., Schmitz C. (2008). Accumulation of Nuclear DNA Damage or Neuron Loss: Molecular Basis for a New Approach to Understanding Selective Neuronal Vulnerability in Neurodegenerative Diseases. DNA Repair.

[165] Cuadrado-Tejedor M., Vilariño M., Cabodevilla F., Del Río J., Frechilla D., Pérez-Mediavilla A. (2011). Enhanced Expression of the Voltage-Dependent Anion Channel 1 (VDAC1) in Alzheimer’s Disease Transgenic Mice: An Insight into the Pathogenic Effects of Amyloid-β. J. Alzheimer’s Dis..

[166] Magrì A., Reina S., De Pinto V. (2018). VDAC1 as Pharmacological Target in Cancer and Neurodegeneration: Focus on Its Role in Apoptosis. Front. Chem..

[167] Driver J.A., Logroscino G., Buring J.E., Gaziano J.M., Kurth T. (2007). A Prospective Cohort Study of Cancer Incidence Following the Diagnosis of Parkinson’s Disease. Cancer Epidemiol. Biomark. Prev..

[168] Meek D.W. (2015). Regulation of the P53 Response and Its Relationship to Cancer. Biochem. J..

[169] Chang J.R., Ghafouri M., Mukerjee R., Bagashev A., Chabrashvili T., Sawaya B.E. (2012). Role of P53 in Neurodegenerative Diseases. Neurodegener. Dis..

[170] Driver J.A., Lu K.P. (2010). Pin1: A New Genetic Link between Alzheimers Disease, Cancer and Aging. Curr. Aging Sci..

[171] Gupta V.K., Scheunemann L., Eisenberg T., Mertel S., Bhukel A., Koemans T.S., Kramer J.M., Liu K.S.Y., Schroeder S., Stunnenberg H.G. (2013). Restoring Polyamines Protects from Age-Induced Memory Impairment in an Autophagy-Dependent Manner. Nat. Neurosci..

[172] Madeo F., Eisenberg T., Pietrocola F., Kroemer G. (2018). Spermidine in Health and Disease. Science (1979).

[173] Gong B., Pan Y., Vempati P., Zhao W., Knable L., Ho L., Wang J., Sastre M., Ono K., Sauve A.A. (2013). Nicotinamide Riboside Restores Cognition through an Upregulation of Proliferator-Activated Receptor-γ Coactivator 1α Regulated β-Secretase 1 Degradation and Mitochondrial Gene Expression in Alzheimer’s Mouse Models. Neurobiol. Aging.

[174] Hou Y., Lautrup S., Cordonnier S., Wang Y., Croteau D.L., Zavala E., Zhang Y., Moritoh K., O’Connell J.F., Baptiste B.A. (2018). NAD^+^ Supplementation Normalizes Key Alzheimer’s Features and DNA Damage Responses in a New AD Mouse Model with Introduced DNA Repair Deficiency. Proc. Natl. Acad. Sci. USA.

[175] Schöndorf D.C., Ivanyuk D., Baden P., Sanchez-Martinez A., De Cicco S., Yu C., Giunta I., Schwarz L.K., Di Napoli G., Panagiotakopoulou V. (2018). The NAD+ Precursor Nicotinamide Riboside Rescues Mitochondrial Defects and Neuronal Loss in IPSC and Fly Models of Parkinson’s Disease. Cell Rep..

[176] Duan W., Mattson M.P. (1999). Dietary Restriction and 2-Deoxyglucose Administration Improve Behavioral Outcome and Reduce Degeneration of Dopaminergic Neurons in Models of Parkinson’s Disease. J. Neurosci. Res..

[177] Andrews Z.B., Diano S., Horvath T.L. (2005). Mitochondrial Uncoupling Proteins in the Cns: In Support of Function and Survival. Nat. Rev. Neurosci..

[178] Howell J.J., Hellberg K., Turner M., Talbott G., Kolar M.J., Ross D.S., Hoxhaj G., Saghatelian A., Shaw R.J., Manning B.D. (2017). Metformin Inhibits Hepatic MTORC1 Signaling via Dose-Dependent Mechanisms Involving AMPK and the TSC Complex. Cell Metab..

[179] Zheng W., Li K., Zhong M., Wu K., Zhou L., Huang J., Liu L., Chen Z. (2024). Mitophagy Activation by Rapamycin Enhances Mitochondrial Function and Cognition in 5×FAD Mice. Behav. Brain Res..

[180] Song Y., Lee W., Lee Y., Kang E., Cha B.-S., Lee B.-W. (2016). Metformin Restores Parkin-Mediated Mitophagy, Suppressed by Cytosolic P53. Int. J. Mol. Sci..

[181] Kocak M., Ezazi Erdi S., Jorba G., Maestro I., Farrés J., Kirkin V., Martinez A., Pless O. (2022). Targeting Autophagy in Disease: Established and New Strategies. Autophagy.

[182] Antico O., Thompson P.W., Hertz N.T., Muqit M.M.K., Parton L.E. (2025). Targeting Mitophagy in Neurodegenerative Diseases. Nat. Rev. Drug Discov..

[183] Lonskaya I., Hebron M.L., Desforges N.M., Schachter J.B., Moussa C.E.-H. (2014). Nilotinib-Induced Autophagic Changes Increase Endogenous Parkin Level and Ubiquitination, Leading to Amyloid Clearance. J. Mol. Med..

[184] Fowler A., Torres-Yhagi Y., Pagan F., Hebron M., Willmarth B., Arellano J., Howard H., Matar S., Chiu T., Ahn J. (2020). 4564 Nilotinib Alters MicroRNAs That Regulate Specific Autophagy and Ubiquitination Genes in the Cerebrospinal Fluid of Parkinson’s Patients. J. Clin. Transl. Sci..

[185] Hebron M.L., Lonskaya I., Moussa C.E.-H. (2013). Nilotinib Reverses Loss of Dopamine Neurons and Improves Motor Behavior via Autophagic Degradation of -Synuclein in Parkinson’s Disease Models. Hum. Mol. Genet..

[186] La Barbera L., Vedele F., Nobili A., Krashia P., Spoleti E., Latagliata E.C., Cutuli D., Cauzzi E., Marino R., Viscomi M.T. (2021). Nilotinib Restores Memory Function by Preventing Dopaminergic Neuron Degeneration in a Mouse Model of Alzheimer’s Disease. Prog. Neurobiol..

[187] Dong Y., Zhang X. (2024). Targeting Cellular Mitophagy as a Strategy for Human Cancers. Front. Cell Dev. Biol..

[188] Chen L., Liu L., Li Y., Gao J. (2018). Melatonin Increases Human Cervical Cancer HeLa Cells Apoptosis Induced by Cisplatin via Inhibition of JNK/Parkin/Mitophagy Axis. In Vitro Cell Dev. Biol. Anim..

[189] Ma M., Lin X., Liu H., Zhang R., Chen R. (2020). Suppression of DRP1-mediated Mitophagy Increases the Apoptosis of Hepatocellular Carcinoma Cells in the Setting of Chemotherapy. Oncol. Rep..

[190] Zhou J., Li G., Zheng Y., Shen H.-M., Hu X., Ming Q.-L., Huang C., Li P., Gao N. (2015). A Novel Autophagy/Mitophagy Inhibitor Liensinine Sensitizes Breast Cancer Cells to Chemotherapy through DNM1L-Mediated Mitochondrial Fission. Autophagy.

[191] Chen Y., Chen H.-N., Wang K., Zhang L., Huang Z., Liu J., Zhang Z., Luo M., Lei Y., Peng Y. (2019). Ketoconazole Exacerbates Mitophagy to Induce Apoptosis by Downregulating Cyclooxygenase-2 in Hepatocellular Carcinoma. J. Hepatol..

[192] Boyle K.A., Van Wickle J., Hill R.B., Marchese A., Kalyanaraman B., Dwinell M.B. (2018). Mitochondria-Targeted Drugs Stimulate Mitophagy and Abrogate Colon Cancer Cell Proliferation. J. Biol. Chem..

[193] Yin O.Q.P., Wang Y., Schran H. (2008). A Mechanism-Based Population Pharmacokinetic Model for Characterizing Time-Dependent Pharmacokinetics of Midostaurin and Its Metabolites in Human Subjects. Clin. Pharmacokinet..

[194] Zhang C., Liu Z., Bunker E., Ramirez A., Lee S., Peng Y., Tan A.-C., Eckhardt S.G., Chapnick D.A., Liu X. (2017). Sorafenib Targets the Mitochondrial Electron Transport Chain Complexes and ATP Synthase to Activate the PINK1–Parkin Pathway and Modulate Cellular Drug Response. J. Biol. Chem..

[195] Llovet J.M., Ricci S., Mazzaferro V., Hilgard P., Gane E., Blanc J.-F., de Oliveira A.C., Santoro A., Raoul J.-L., Forner A. (2008). Sorafenib in Advanced Hepatocellular Carcinoma. N. Engl. J. Med..

[196] Yu L., Wang C., Pan F., Liu Y., Ren X., Zeng H., Shi Y. (2019). HePTP Promotes Migration and Invasion in Triple-Negative Breast Cancer Cells via Activation of Wnt/β-Catenin Signaling. Biomed. Pharmacother..

[197] Zheng P., Li J., Kros J.M. (2018). Breakthroughs in Modern Cancer Therapy and Elusive Cardiotoxicity: Critical Research-practice Gaps, Challenges, and Insights. Med. Res. Rev..

[198] Chen D., Zhang T., Lee T.H. (2020). Cellular Mechanisms of Melatonin: Insight from Neurodegenerative Diseases. Biomolecules.

[199] Li Y., Li S., Zhou Y., Meng X., Zhang J.-J., Xu D.-P., Li H.-B. (2017). Melatonin for the Prevention and Treatment of Cancer. Oncotarget.

[200] Verhoef B.-E., Maunsell J.H. (2016). Attention Operates Uniformly throughout the Classical Receptive Field and the Surround. eLife.

[201] Bouadi O., Tay T.L. (2021). More Than Cell Markers: Understanding Heterogeneous Glial Responses to Implantable Neural Devices. Front. Cell Neurosci..

[202] Andreux P.A., Blanco-Bose W., Ryu D., Burdet F., Ibberson M., Aebischer P., Auwerx J., Singh A., Rinsch C. (2019). The Mitophagy Activator Urolithin A Is Safe and Induces a Molecular Signature of Improved Mitochondrial and Cellular Health in Humans. Nat. Metab..

[203] Riley R.T., Torres O., Showker J.L., Zitomer N.C., Matute J., Voss K.A., Gelineau-van Waes J., Maddox J.R., Gregory S.G., Ashley-Koch A.E. (2012). The Kinetics of Urinary Fumonisin B_1_ Excretion in Humans Consuming Maize-based Diets. Mol. Nutr. Food Res..

[204] Guthrie A.R., Chow H.-H.S., Martinez J.A. (2017). Effects of Resveratrol on Drug- and Carcinogen-Metabolizing Enzymes, Implications for Cancer Prevention. Pharmacol. Res. Perspect..

[205] London C.A., Malpas P.B., Wood-Follis S.L., Boucher J.F., Rusk A.W., Rosenberg M.P., Henry C.J., Mitchener K.L., Klein M.K., Hintermeister J.G. (2009). Multi-Center, Placebo-Controlled, Double-Blind, Randomized Study of Oral Toceranib Phosphate (SU11654), a Receptor Tyrosine Kinase Inhibitor, for the Treatment of Dogs with Recurrent (Either Local or Distant) Mast Cell Tumor Following Surgical Excision. Clin. Cancer Res..

[206] Zlotorynski E. (2016). Quad-Jumping. Nat. Rev. Mol. Cell Biol..

[207] Du Y., Zhu Y.-J., Zhou Y.-X., Ding J., Liu J.-Y. (2022). Metformin in Therapeutic Applications in Human Diseases: Its Mechanism of Action and Clinical Study. Mol. Biomed..

[208] Gao F., Xu T., Zang F., Luo Y., Pan D. (2024). Cardiotoxicity of Anticancer Drugs: Molecular Mechanisms, Clinical Management and Innovative Treatment. Drug Des. Devel Ther..

[209] Martens C.R., Denman B.A., Mazzo M.R., Armstrong M.L., Reisdorph N., McQueen M.B., Chonchol M., Seals D.R. (2018). Chronic Nicotinamide Riboside Supplementation Is Well-Tolerated and Elevates NAD+ in Healthy Middle-Aged and Older Adults. Nat. Commun..

[210] Conze D., Crespo-Barreto J., Kruger C. (2016). Safety Assessment of Nicotinamide Riboside, a Form of Vitamin B_3_. Hum. Exp. Toxicol..

[211] Harron D.W.G. (1992). Distinctive Features of Rilmenidine Possibly Related to Its Selectivity for Imidazoline Receptors. Am. J. Hypertens..

[212] Li M.-M., Teng J., Wang Y. (2021). Chronic Colchicine Poisoning with Neuromyopathy, Gastric Ulcers and Myelosuppression in a Gout Patient: A Case Report. World J. Clin. Cases.

[213] Choi S., Lee Y.J., Jeong J.H., Jung J., Lee J.W., Kim H.J., Ko B.S., Son B.H., Ahn S.H., Lee Y. (2021). Risk of Endometrial Cancer and Frequencies of Invasive Endometrial Procedures in Young Breast Cancer Survivors Treated With Tamoxifen: A Nationwide Study. Front. Oncol..

